# Deep-Sea, Deep-Sequencing: Metabarcoding Extracellular DNA from Sediments of Marine Canyons

**DOI:** 10.1371/journal.pone.0139633

**Published:** 2015-10-05

**Authors:** Magdalena Guardiola, María Jesús Uriz, Pierre Taberlet, Eric Coissac, Owen Simon Wangensteen, Xavier Turon

**Affiliations:** 1 Department of Marine Ecology, Center for Advanced Studies of Blanes (CEAB-CSIC), Girona, Spain; 2 Université Grenoble Alpes, Laboratoire d’Ecologie Alpine (LECA), F-38000, Grenoble, France; 3 Centre National de la Recherche Scientifique (CNRS), Laboratoire d’Ecologie Alpine (LECA), F-38000, Grenoble, France; 4 Department of Animal Biology, University of Barcelona, Barcelona, Spain; Universite Pierre et Marie Curie, FRANCE

## Abstract

Marine sediments are home to one of the richest species pools on Earth, but logistics and a dearth of taxonomic work-force hinders the knowledge of their biodiversity. We characterized α- and β-diversity of deep-sea assemblages from submarine canyons in the western Mediterranean using an environmental DNA metabarcoding. We used a new primer set targeting a short eukaryotic 18S sequence (ca. 110 bp). We applied a protocol designed to obtain extractions enriched in extracellular DNA from replicated sediment corers. With this strategy we captured information from DNA (local or deposited from the water column) that persists adsorbed to inorganic particles and buffered short-term spatial and temporal heterogeneity. We analysed replicated samples from 20 localities including 2 deep-sea canyons, 1 shallower canal, and two open slopes (depth range 100–2,250 m). We identified 1,629 MOTUs, among which the dominant groups were Metazoa (with representatives of 19 phyla), Alveolata, Stramenopiles, and Rhizaria. There was a marked small-scale heterogeneity as shown by differences in replicates within corers and within localities. The spatial variability between canyons was significant, as was the depth component in one of the canyons where it was tested. Likewise, the composition of the first layer (1 cm) of sediment was significantly different from deeper layers. We found that qualitative (presence-absence) and quantitative (relative number of reads) data showed consistent trends of differentiation between samples and geographic areas. The subset of exclusively benthic MOTUs showed similar patterns of β-diversity and community structure as the whole dataset. Separate analyses of the main metazoan phyla (in number of MOTUs) showed some differences in distribution attributable to different lifestyles. Our results highlight the differentiation that can be found even between geographically close assemblages, and sets the ground for future monitoring and conservation efforts on these bottoms of ecological and economic importance.

## Introduction

The field of biodiversity assessment has been revolutionized in recent years by the application of the genetic barcode concept to DNA extracted from environmental samples and the use of next generation sequencing technologies. The resulting new approach, called DNA metabarcoding [1], was first developed in prokaryote studies and has been successfully applied to studies of microbial eukaryotes and, to a lesser extent, to fungi, plant, and animal communities (reviewed in [2–6]). In particular, metabarcoding has proven useful to target marine eukaryotic communities, both planktonic and benthic (e.g. [7–14]). Metabarcoding has applications not only in biodiversity assessment of marine communities *per se*, but also in studies of ecological impacts (e.g. oil spills: [15,16]), ecosystem dynamics (e.g. [17]), symbioses (e.g. [18,19] diets (e.g. [20]), study of ancient DNA (e.g. [21]) or identification of endangered or pest species (e.g. [22,23]). The spectrum of potential applications of metabarcoding will likely continue to widen [24–26].

Metabarcoding techniques include both species identification from bulk organismal samples where the organisms have been isolated before the analysis, or from environmental DNA (eDNA) defined as the genetic material obtained directly from samples (soil, sediment, water…) without first isolating any target organisms [1, 27]. In a more restricted sense, eDNA is the one obtained from samples without any obvious signs of biological source material [26]. This definition is somewhat contentious, though, as big-size organisms will be present as cellular remains or free DNA; while complete, living small-organisms can be sampled [5,26]. In fact eDNA, as commonly referred to in the literature, is a continuum from DNA contained in whole living organisms to extraorganismal DNA in tissue remains or free, extramembranous DNA in the environment [28]. The mode of extraction may be more relevant than the sampling method in determining which fraction of the DNA is captured. We chose to use a fast extraction method without a lysis step, which implied that our samples recovered mostly extracellular DNA. This method was recently developed for soil samples [29]. As extracellular DNA adsorbed to particulate matter persists much longer than free DNA in water [5, 21, 28, 30], targeting this fraction will likely allow buffering short-term spatial and temporal heterogeneity [29].

Metabarcoding studies target DNA that may be old and degraded, thus requiring short barcode sequences [1, 31]. In metabarcoding, the universality of the primers is crucial [32] and, due to the short length of the DNA amplified, identification to species level is not always possible. The identification of Molecular Operational Taxonomic Units (MOTUs), even without a species name, suffices for many ecological applications. The ability to characterize the biodiversity of a sample in this way outcompetes the classical use of indicator species (which are often biased towards emblematic or apparent species [4, 33]) or the traditional taxonomic studies where only a fraction of the present biodiversity can be assessed, in a time-consuming and costly process [2, 4, 26] depending heavily on available taxonomic expertise (which is scarce worldwide, particularly for invertebrates, [34]). The study of environmental DNA is by now a “game-changer” in the way we assess and monitor biodiversity [35].

The marine sediments are allegedly home to one of the richest species pools on Earth, but logistics and a dearth of taxonomic work-force hinder the knowledge of their biodiversity, and more so for deep-sea sediments [36–40]. These communities have key ecological roles, provide important ecosystem services, and are sensitive to anthropogenic disturbances [37, 40–42]. They also provide a field laboratory for analysing the link of biodiversity patterns above and below the sediment-water interface, which is poorly understood [43]. Deep-sea sediment communities are one clear example where metabarcoding techniques can foster a leap forward in our ability to describe biodiversity patterns and dynamics [44,45] and indeed they have been the target of studies using eukaryote DNA obtained from environmental samples(e.g. [5,10, 39, 46, 47]).

In this study we analysed the DNA present in sediments from deep-sea submarine canyons in the Western Mediterranean. These ecosystems represent hotspots of biodiversity and are important drivers of the dynamics of the commercial fisheries associated with them [41, 48]. These assemblages face important threats nowadays in the Mediterranean, mostly related to human activities [48–50]. We wanted to test a metabarcoding approach targeting environmental DNA (enriched in extracellular DNA) from sediment corers; amplifying short sequences of the 18S rRNA gene, and sequencing them on an Illumina platform. We analysed the sequence data for spatial heterogeneity at several scales, compared information from layers of sediment, and assessed patterns of distribution with depth. Our ultimate goal was to develop efficient tools to characterise patterns of community structure that could set the ground for monitoring studies on these bottoms of ecological and economic value.

## Material and Methods

### Sampling

The samples were collected in the Western Mediterranean during the DOSMARES and INDEMARES cruises aboard the oceanographic vessel R/V García del Cid of the Spanish Research Council in March and June, 2012, respectively (S1 Table). The DOSMARES project focused on the Blanes Canyon (NE Iberian Peninsula) and the adjacent open slope area (S1 Fig). Three zones were sampled in the INDEMARES project: the Cap de Creus Canyon in the NE Iberian Peninsula, and the Menorca Canal and the Serra de Tramuntana Slope in the Balearic Islands (S1 Fig). In both locations, permits for the sampling activities were issued by the Spanish Ministry of Agriculture, Food, and the Environment (MAGRAMA). We will hereafter use the term Zone to refer to the five major study points (CC: Cap de Creus, BC: Blanes Canyon, OS: Blanes Open Slope, MC: Menorca Canal, ST: Serra de Tramuntana Slope). We will use the term Area to refer to the Iberian Peninsula or the Balearic Islands coasts. Geophysical features of the sampled zones are described in [48, 51–54].

Samples were taken either with a multicorer (DOSMARES) or a box corer (INDEMARES) and then sub-sampled with mini-corers 3.6 cm in diameter to get 5 cm of sediment thickness. For the DOSMARES project, the mini-corers were further split into three layers (A: first cm; B: second cm; C: third to fifth cm); for the INDEMARES project the samples were not separated by layer. Two types of replication were used: in the INDEMARES project, three mini-corers were obtained from the same haul (i.e., from the same box corer), while in the DOSMARES project one mini-corer each was collected from two hauls obtained in the same locality (separated ca. 100 m). All samples were then preserved in ethanol, although one of the replicates per locality of the DOSMARES project was preserved in DESS (20% DMSO; 0.25 M EDTA; NaCl saturated, pH = 8) [55] as they were originally intended also for morphological analyses. A total of 81 samples from 20 localities were obtained, 51 from the DOSMARES cruise comprising depths of between 500 and 2,250 m, and 30 from the INDEMARES cruise at depths of between 100 and 800 m. S1 Table gives the particulars of the different localities sampled.

### DNA extraction, amplification and next generation sequencing

The sediment of each sample was homogenized and 9 grams of sediment were processed with a protocol optimized for the extraction of extracellular DNA (as in [29]). In short, the sediment was mixed with an equivalent volume of phosphate buffer (Na_2_HPO_4_/NaH_2_PO_4_; 0.12 M, pH ≈ 8) and the mixture was then homogenized in a shaker for 15 minutes. This step allows recovery of the DNA adsorbed to particulate matter, while intraorganismal DNA is mostly avoided [29]. An aliquot (2 ml) was centrifuged for 10 min at 10000 rcf, and 0.5 ml of the supernatant containing extracellular DNA was extracted using DNeasy Blood Tissue Kit from Qiagen. One sample of layer B in the Blanes Canyon was excluded from the analyses because it had less than 9 grams due to a problem during processing.

A hypervariable fragment of the 18S rRNA gene in the v7 region (usually 100–110 bp) was amplified with a new universal primer pair for eukaryotes designed by PT and EC using the ecoPrimers program [32]. This program optimizes the primers by taking into account both the conservation of the primers' targets and the variability of the amplified region. The primer pair was labelled 18S_allshorts (Forward 5’-TTTGTCTGSTTAATTSCG-3’ and Reverse 5’-GCAATAACAGGTCTGTG-3’). Primer logos [56] and other parameters reflecting the specificity and suitability of these primers for eukaryotes are presented in S2 Fig. Amplification was performed in a total volume of 30 μl with 0.24 μl of AmpliTaq® Gold DNA polymerase (Applied Biosystems) 5U/μl, 1.2 μl of 5 μM of forward and reverse primers mix, 3 μl of buffer 10x, 3 μl of MgCl2, 2.4 μl dNTP (2.5 mM each), 0.24 μl of BSA (20mg/ml) and 3 μl of DNA template. The PCR conditions consisted in a first denaturation step of 10 min at 95°C and then 45 cycles of denaturation at 95°C for 30 s, hybridisation at 45°C for 30 s and elongation at 72°C for 30 s.

Three PCR per sample were performed and pooled. Tags of 8 base pairs were added to the forward and reverse primers to uniquely label each sample. The tags were created with the program oligotag of the OBITools software [57] (http://metabarcoding.org/obitools) and had at least 3 different base pairs between them. The amplification products were purified with the MiniElute PCR Purification Kit (Qiagen) and the DNA was quantified by the QIAxcel device. 14 negative controls were run amplifying ultrapure water (Milli-Q system). Library preparation (a single library was generated) and sequencing (MiSeq Illumina platform, 2x150 bp paired-ends partial run) were performed at the FASTERIS facilities (Plan-les-Quates, Switzerland; https://www.fasteris.com/dna/).

### Read filtering and taxon assignment

The sequence reads were analysed using the OBITools software [57]. First, the paired ends of each sequence were assembled. Exact matches of sample tags were then used to assign reads to samples, and low-quality sequences (fastq average quality score<35) and sequences of non-suitable length (<80 bp) were removed. Strictly identical sequences were dereplicated and assigned a count number per sample. Rare sequences (i.e., sum of counts<10) were eliminated. The obiclean program was then run to detect amplification/sequencing errors and chimeric sequences. This denoising procedure gives each sequence within a PCR product the status of ‘‘head” (most common sequence among all sequences that can be linked with a single indel or substitution), ‘‘singleton” (no other variant with a single difference in the relevant PCR product), or ‘‘internal” (all other sequences not being ‘‘head” or ‘‘singleton”, i.e. most likely corresponding to amplification/sequencing errors) [57]. Only sequences that were more often ‘head’ or ‘singleton’ than ‘internal’ in the global dataset were kept for the following steps. The resulting dataset was further checked with UCHIME [58], using both *de novo* and reference-based searches, and no remaining chimeric sequences were detected.

The sequences were then clustered (heuristic clustering in sumaclust program) using a cut-off value of 96% sequence similarity. The rationale for the choice of the threshold value is presented in S1 File. For each cluster, the sequence with the higher number of reads was taken as the cluster representative for further analyses, and the counts of the remaining sequences were added to it. The taxonomic assignment was performed using the ecotag program [59] which finds similar sequences in a reference database and assigns the query sequence to their most recent ancestor using the NCBI taxonomy database [60]. The reference database was constructed with the program ecoPCR [61] for the 18S fragment based on the release 117 of the EMBL database. Only the sequences with a best identity match of 90% or more were kept at this step. This filtering would remove any remaining PCR artefacts, but at the cost of losing organisms from deep branches or those not adequately represented in reference databases. The trade-off between excluding artefacts and genuine, albeit rare, sequences is not easily solved [62]. Our 90% threshold represents a conservative approach, following other studies in benthic communities (e.g., [2, 8, 9,15]). In the interval 90–100%, the frequency of MOTUs showing a given similarity was quite evenly distributed, with an increase at similarities ≥98%, which made up for 25% of all MOTUs (S3 Fig).

Once the taxa list was acquired, further filtering processes were carried out to refine the dataset. We set to 0 the counts of sequences per sample that might correspond to samples cross-contamination (due to the tagging system used to identify the samples, and to the library preparation step before sequencing): for each sequence the counts per sample were ordered from lowest to highest and those corresponding to a cumulative frequency inferior to 0.01 were set to 0. Second, sequences present in the negative controls after the previous step were removed. Finally, we manually reviewed retained sequences and eliminated clearly non-marine organisms (these could be contaminations or DNA of continental origin present in the sediment). The final retained sequences were considered as molecular operational taxonomic units (MOTUs) on which we performed the analyses. These sequences, as well as the OBITools commands used, have been uploaded to the DRYAD repository (doi: 10.5061/dryad.520gq)

For the analysis of distribution patterns, we grouped our sequences following the major Super-Groups of eukaryotes suggested by [63], with one exception: we split the Super-Group Opisthokonta into Metazoa, Fungi, and other Opisthokonta. As we were particularly interested in the Metazoa, we also analysed their abundance data by phylum.

### Data analysis

A table was constructed with the number of reads of each MOTU per sample. This dataset was used both qualitatively and quantitatively. A similarity index based on presence-absence data (Jaccard index) was obtained with the Primer v6 statistical package [64]. Quantitative inference using number of reads of a given MOTU is controversial [5, 65, 66] and it has been suggested that relative abundances, rather than absolute values, are more reliably obtained from metabarcoding data [6, 13]. We therefore calculated the relative number of reads of each MOTU per sample, and averaged these values over samples to obtain a measure of global relative abundance for each MOTU or group of MOTUs. The relative number of reads was square-root transformed and used to calculate a quantitative similarity matrix using the Bray-Curtis index in Primer v6. To assess the small-scale heterogeneity of the samples, we compared the similarities found with both qualitative and quantitative data using different groupings of samples. Thus, we compared similarities within the same box-corer in the INDEMARES project (three replicates from the same haul) with the ones found in the DOSMARES samples (two independent hauls from the same locality), and with the similitudes obtained when comparing samples from different localities in the same zone (CC, BC, OS, MC, ST), different zones within the same area (Iberian Peninsula and Balearic Islands), and different areas.

Rarefaction curves were obtained with the vegan 2.0–7 package for R [67], using function *rarecurve*, to assess the gain in MOTU richness as we increase the number of reads for each sample. We also used the *specaccum* function, using random addition of samples and 1000 permutations, to investigate the relationship of MOTU richness with increasing numbers of samples.

We also analysed the geographic span of the different MOTUs in terms of the number of localities and zones where a given MOTU is present. MOTU richness (rarefied to the number of reads corresponding to the sample with less reads to allow for statistical comparisons) was also calculated using the function DIVERSE of Primer v6. These values were compared among zones (all samples), across layers of sediment (DOSMARES samples), and among depths (DOSMARES samples).

Permutational analyses of variance were performed with the Windows PERMANOVA module [68] incorporated in Primer v6. We first combined the DOSMARES and INDEMARES samples to test the effect of zone (fixed factor with five levels: BC, OS, CC, MC, ST) and locality (nested within zone). PERMANOVA analyses were also performed for the DOSMARES samples alone to test the effect of layer (fixed, with three levels: first cm, second cm, and rest of the sample) and depth (fixed, 7 levels). For all significant factors, permutational pair-wise tests were performed. As these tests were uncorrected for multiple comparisons, we applied a Benjamini-Yekutieli FDR correction following Narum [69]. Tests of multivariate dispersions (PERMDISP function) were also done to ascertain whether significant values in PERMANOVA were a result of different heterogeneity of the groups (spread) instead of different multivariate mean location.

Results were also visualized with non-metric multidimensional scaling (nmMDS) ordinations. The analyses were performed in vegan [67] using the *metaMDS* function. Unlike MDS programs that find a single configuration by iteration and thus can get trapped in local optima, *metaMDS* performs different random starts and compares them to find a stable solution. We set the number of random starts to 500. We performed the nmMDS ordinations on distance matrices based on the Jaccard coefficient (in distance form) using presence-absence data, and on matrices based on the Bray Curtis index (in distance form) taking into account relative abundances. MDS configurations were obtained for the different localities studied (both projects pooled) and, for the DOSMARES project alone, for the different layers of sediment and the different depths sampled. The latter analysis was performed separately for the first layer of sediment and for the other two layers pooled (following PERMANOVA results, see below). The different configurations obtained with presence-absence and relative abundance data were compared using Procrustes analyses [70] as implemented in vegan (function *Protest*). Function *envfit* of vegan was used to correlate depth with the ordinations obtained for the two sediment layers considered, and to obtain and plot the corresponding gradient vectors in nmMDS ordinations.

Additionally, a Mantel test was conducted to check for correlations between geographic distances among localities (in kilometres) and MOTU dissimilarity using the Jaccard index (transformed to distance). The Mantel test was performed using the ade4 package for R (function *mantel*.*rtest*) and its significance was tested by permutation [71].

To gain insights into the ecological function of the main groups of organisms, a separate analysis was performed using only MOTUs that could be assigned to benthic groups. We considered as benthic MOTUs those with a known holobenthic cycle or, if pelago-benthic, with restricted dispersal phase (lecitotrophic larvae). This classification was based on an exhaustive perusal of available literature on the closest GenBank matches of each MOTU and on general characteristics of the group (Class, Family, Genus) where the MOTU belongs. The selected MOTUs are indicated in the deposited database (doi: 10.5061/dryad.520gq). In addition, we ran separate analyses for the three most MOTU-rich metazoan phyla (Annelida, Arthropoda, and Nematoda, see Results).

## Results

The MiSeq partial run produced a total of 3,840,493 reads. After sample assignment, quality and sequence-length filtering, and elimination of rare sequences, we were left with 2,720,318 reads corresponding to 12,751 different sequences. Further sequence error pruning and chimera removal (obiclean) reduced the number of sequences finally retained to 8,215. These sequences were clustered (96% similarity cut-off) and, after final checking, 1,629 MOTUs with >90% sequence identity to their best hit were retained. They belonged to 10 eukaryote Super-Groups (modified from [63], see Methods). The mean number of reads and MOTUs per sample were 11,412±90.51 and 221.44±11.69 (mean±SE). The controls featured less than 750 reads per sample on average. The list of the MOTUs considered, their taxonomic assignment, and the number of reads in the samples is available in the Dryad repository (doi:10.5061/dryad.520gq).

**Fig 1:**
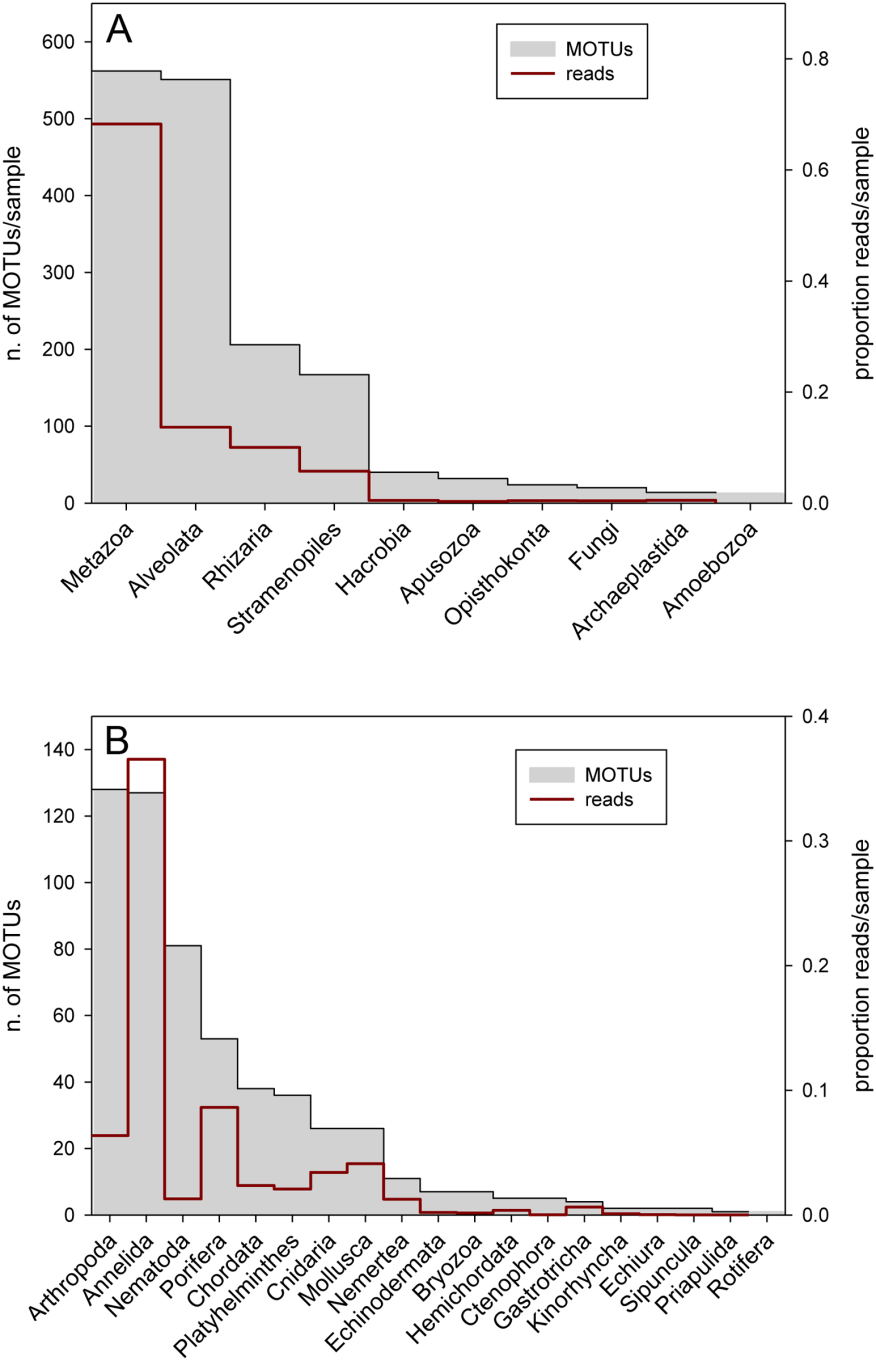
Rank order of the main Super-Groups (A) and metazoan phyla (B) according to total number MOTUs in the samples. The red line indicates the mean proportion of reads of the taxa in the samples.

The ranking of the different Super-Groups, as per total number of MOTUs, is plotted in Fig 1. The relative number of reads per sample is also represented. Metazoa was the most abundant group for both variables. However, they had a disproportionally higher abundance when considering relative read numbers, likely due to their bigger sizes which translate into higher ribosomal DNA presence in the sediment environment. Alveolata (mostly dinoflagellates and ciliates), Rhizaria and Stramenopiles were the next most abundant groups (again both in terms of total MOTUs and relative abundance, Fig 1). The other Super-Groups had a marginal representation in our samples. The proportion of MOTUs of the different Super-groups as per sampling locality is shown in S4 Fig.

Within metazoans, the most MOTU-rich phylum was Arthropoda (Crustacea), followed by Annelida (Polychaeta) and Nematoda. Porifera, Chordata, Playthelminthes, Cnidaria and Mollusca followed at some distance (Fig 1). These phyla were consistently the most diverse in all localities (S4 Fig). The differences between richness and relative abundances of the phyla were marked. While annelids ranked second in number of MOTUs, they dominated when considering proportion of reads. Likewise, Nematoda had relatively few numbers of reads even if it was one of the most diverse groups. These differences correlate with size differences of the taxa and suggest that relative number of reads may capture relative biomass of the groups.

### Beta-diversity and rarefaction analyses

**Fig 2:**
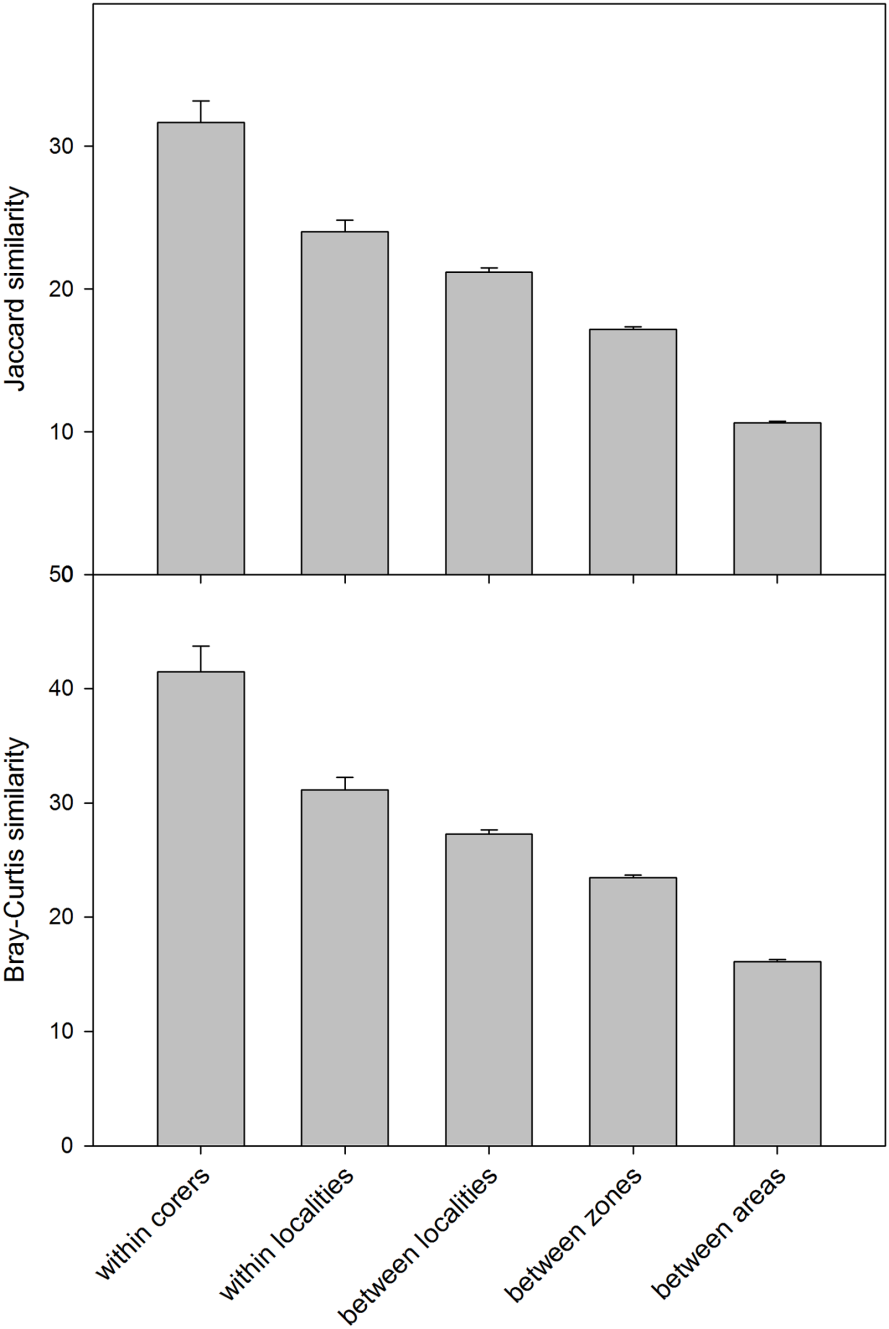
Average similarities between pairs of samples in different categories of comparisons. Both the Jaccard index (presence-absence data) and the Bray-Curtis index (relative abundance data) results are presented. Error bars are standard errors.

The values of compositional similarity (Jaccard index) based on presence-absence data between replicate samples (i.e., individual mini-corers) taken from the same box-corer (INDEMARES project) were of 31.67±1.49% (mean±SE). Similarities between two consecutive hauls in the same locality (DOSMARES project) were lower (24.02±0.79%). The mean similarity decreased progressively (Fig 2) as we compared samples from localities in the same zone, samples from localities from different zones within the same general area (Iberian Peninsula or Balearic Islands) and, finally, samples from localities from different areas, which presented a low similarity (only 10.63±0.13% shared MOTUs). The major break in species replacement occurred between the two geographic areas considered, as the number of shared MOTUs between any two samples decreased ca. 40% with respect to the number found when comparing samples within areas (Fig 2). A similar pattern was found when comparing similarities based on relative abundance (Bray-Curtis index), which is highest (41.50±2.25%) among replicates within box-corers and lowest (16.09±0.22%) when comparing samples from different areas (Fig 2). All these mean similarities were significantly different among all groupings for both indexes (ANOVA test, p<0.001, and Student-Newman-Keuls post-hoc tests, all p<0.05).

**Fig 3:**
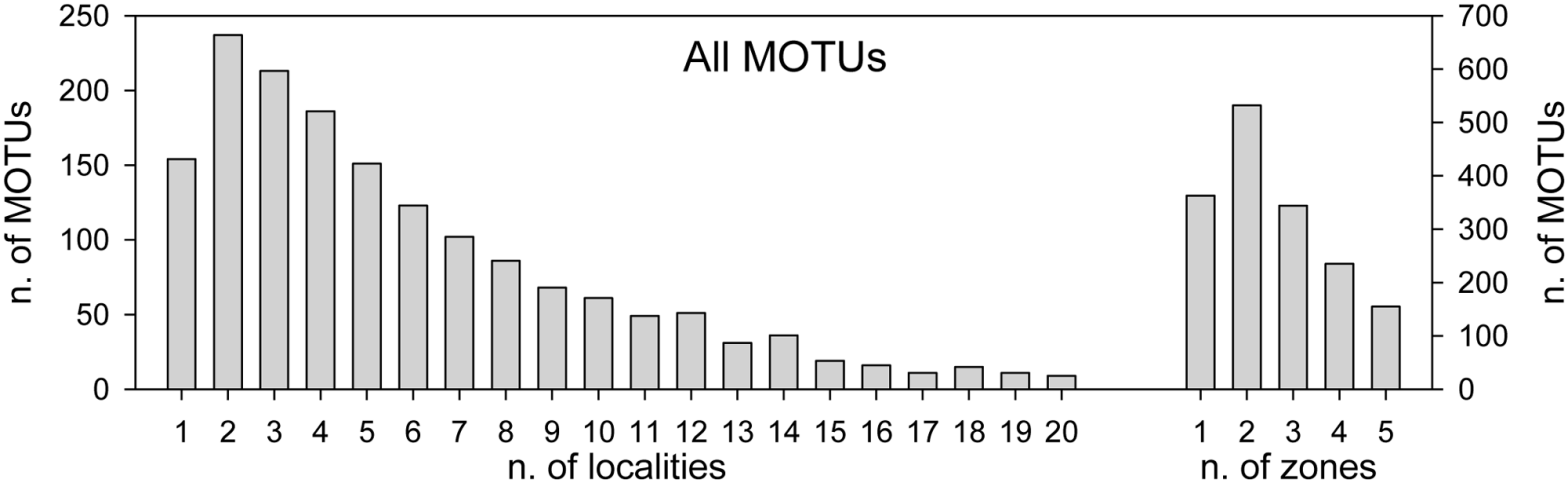
Distributional span of the MOTUs. The graph represents the frequency histograms of the number of MOTUs present in 1 to 20 samples or in 1 to 5 zones (right axis).

We also examined the distributional span of the MOTUs recovered, in terms of the number of localities (out of 20 localities) or number of zones (CC, BC, OS, MC, ST) where a particular MOTU is present. The frequency distribution of numbers of localities (Fig 3) showed a peak at two localities, with a long right tail (median: 4 localities, mean: 5.88 localities). Only 9 MOTUs were found in all 20 samples, these were three polychaetes, two dinoflagellates, two sponges, a radiolarian, and an ascidian. When considering zones, MOTUs present in 2 zones were the dominant (Fig 3).

For each particular sample we could compute rarefaction curves relating number of reads with MOTU richness (S5 Fig). The results showed that only the samples with higher number of reads tended to reach a plateau in the number of MOTUs. The remaining samples reached a zone of slope change but not a saturation zone. This indicates a high complexity of the communities, so that the sequencing depth attained (mean >11,000 reads/sample) captured a significant amount of the diversity present, but an exhaustive record would need a higher coverage.

Curves of species (MOTUs) accumulation, obtained through random (100 replicates) addition of samples, showed that the slope change occurs when pooling between 20 and 30 samples (S6 Fig), again indicating a noticeable heterogeneity in species composition in our samples.

### Community structure

**Fig 4:**
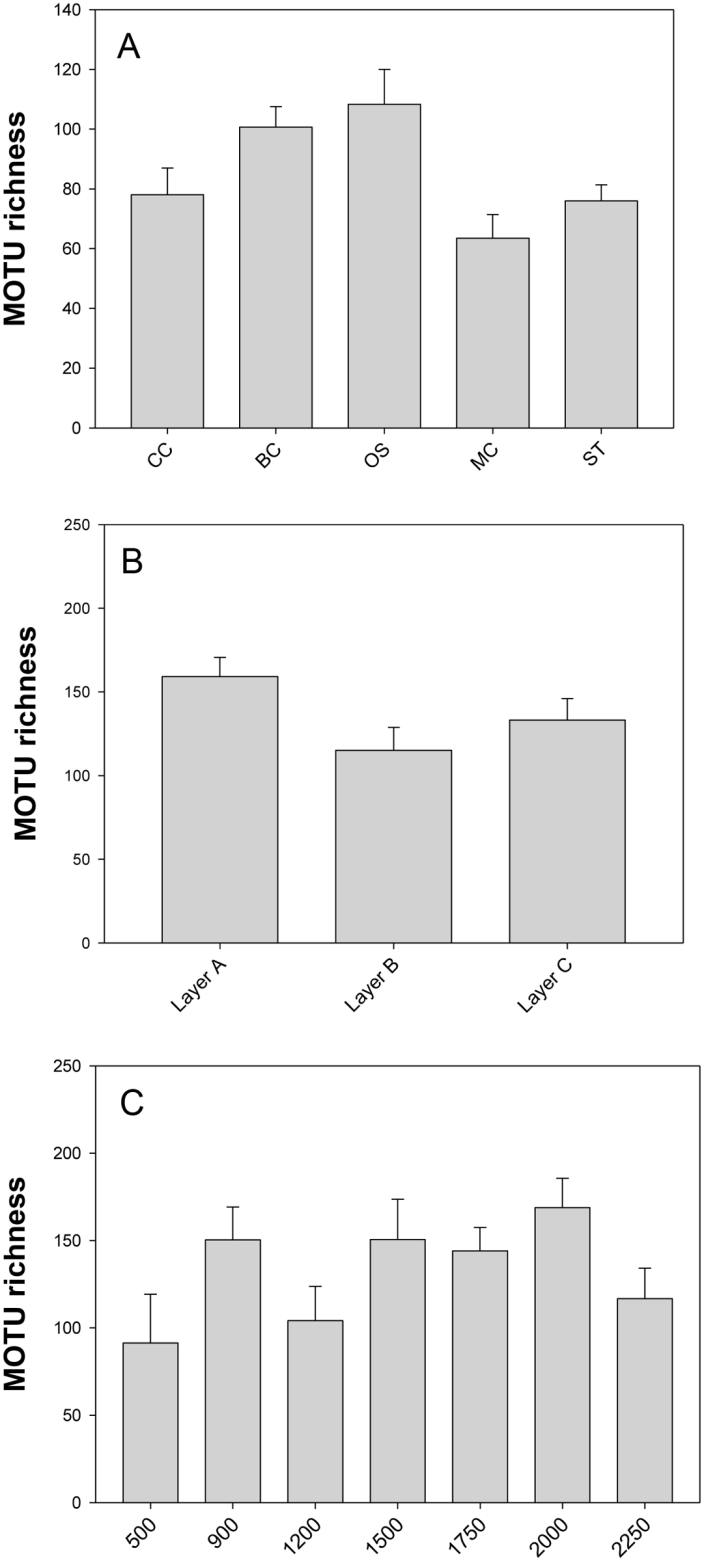
MOTU richness. Values rarefied to the number of reads of the smallest sample in each grouping are given for the different zones (A), layers (B), and depths (C) considered. Values are sample means and error bars are standard errors.

For statistical comparison, values of MOTU richness were rarefied to the number of reads of the sample with the least of them (which varies according to the groupings made). These values were then compared between zones, sediment layers (DOSMARES project) and depths (DOSMARES project) (Fig 4). As per zones, rarefied MOTU richness (averaged over samples) was between a mean of 108 (Open Slope) and 63 (Menorca Canal). The mean value was significantly lower in the Menorca Canal than in the Blanes Canyon and its Open Slope (ANOVA p = 0.014, followed by Student-Newman-Keuls test). Layer A was the most MOTU-rich (average 159), but the differences were not significant (Kruskal-Wallis test, p = 0.086, Fig 4). Likewise, no significant differences were found between depths in the Blanes area (ANOVA, p = 0.069, Fig 4), albeit these values were the lowest at the shallowest samples (500 m). An analysis of an index that takes into account the relative abundance (in number of reads) of MOTUs, the Simpson index, showed the same overall trends as the qualitative MOTU richness index, except that there was no significant difference among zones (results not shown). Finally, a comparison of MOTU richness and the Simpson index of the replicates preserved in DESS and in ethanol (DOSMARES project) showed no significant differences (paired *t*-tests, all p>0.5).

**Fig 5:**
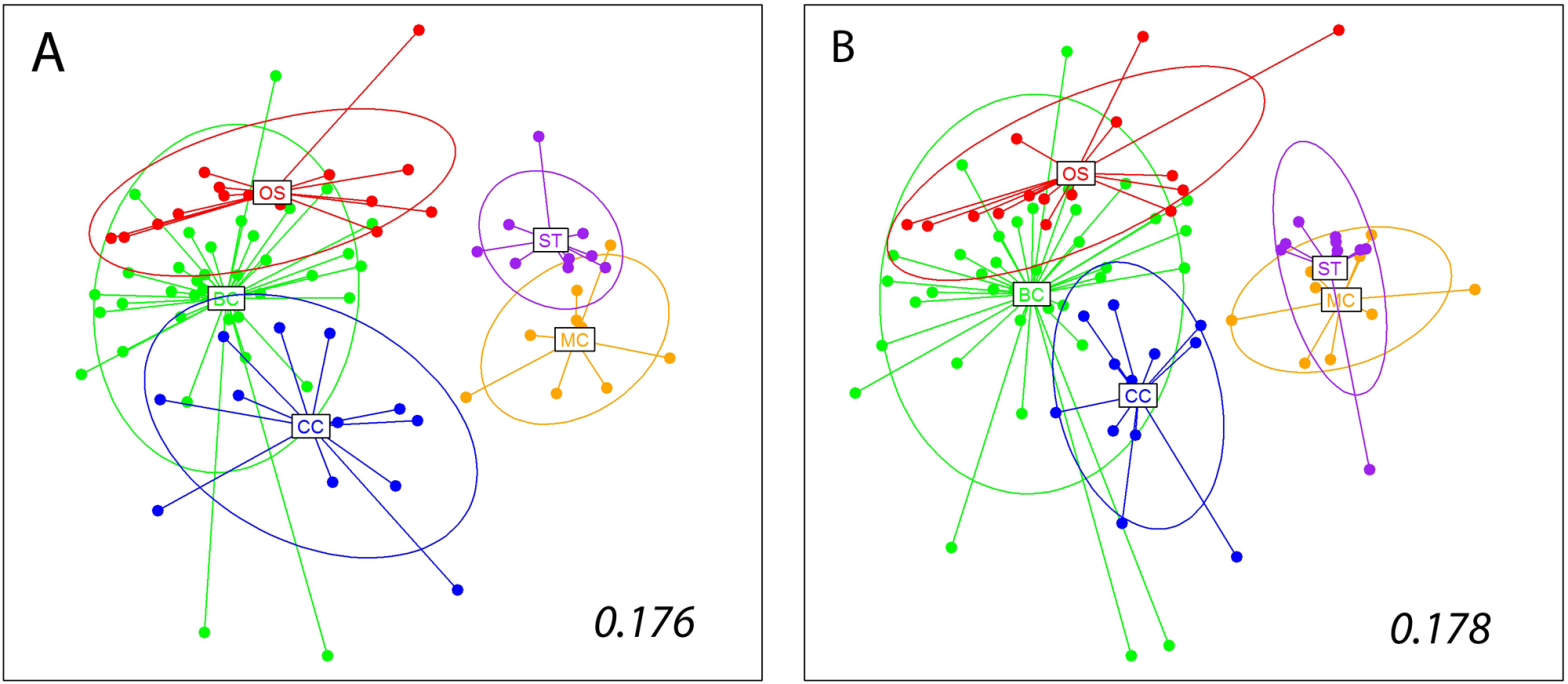
Non-metric Multidimensional Scaling plots of the samples obtained using Jaccard dissimilarity index (A), and Bray-Curtis dissimilarity index (B) for the whole dataset. The centroids for the different zones, and the corresponding inertia ellipses, are indicated. Numbers inside the plots are the stress values of the retained configurations.

The results of the nmMDS using the Jaccard index showed a clear distinction between zones situated in the Iberian Peninsula and in the Balearic Islands (Fig 5A). Within these two areas, the different zones were also distinguished, albeit with some overlap of the inertia ellipses (particularly between the Blanes Canyon and the adjacent Open Slope). PERMANOVA analyses showed that all zones were different from one another in terms of community composition (Table 1), except for the comparison between Menorca Canal and Serra de Tramuntana Slope, which was not significant after correction for multiple comparisons. PERMDISP detected differences in heterogeneity levels between zones that could explain some of the pairwise differences found (Table 1). The nested factor locality was also significant, indicating relevant within zone variability, as well as heterogeneity of dispersion at this level (Table 1). The nmMDS representation using quantitative (Bray-Curtis index) data showed a similar sample ordination (Fig 5B) than the one obtained with qualitative data (Procrustes correlation = 0.991, p<0.001). Likewise, statistical analyses gave essentially the same outcomes for quantitative and qualitative data, and the latter are not shown (the only difference being that the pairwise comparison between Blanes Canyon and the adjacent Open Slope was not significant with quantitative data).

**Table 1:** PERMANOVA analysis (9,999 permutations) comparing the five zones surveyed using Jaccard index. PERMDISP probabilities for homogeneity of dispersion are also shown. Pairwise tests for levels of the factor Zone are presented with uncorrected P-values. Significant values after FDR correction are indicated with an asterisk. The symbol § denotes comparisons for which PERMDISP detected a significant heterogeneity of dispersion.

	*df*	*SS*	*pseudo-F*	*P-value*	*Permdisp*
Zone	4	53,130	3.357	<0.001	<0.006
Locality(Zone)	15	62,097	1.519	<0.001	<0.001
Residual	61	166,230			
**Zone**					
Comparison	*t*	*P-value*			
BC- CC	1.6391	<0.001*			
BC- MC	2.2249	<0.001*^§^			
BC- OS	1.2851	0.008*			
BC- ST	2.2953	<0.001*^§^			
CC- MC	1.6613	0.006*			
CC- OS	1.6176	0.001*			
CC- ST	1.8087	0.003*			
MC- OS	2.0085	<0.001*			
MC- ST	1.4561	0.035			
OS- ST	2.0636	<0.001*^§^			

The Mantel test comparing geographic distances with community dissimilarities using the Jaccard index (transformed to distances) showed a significant outcome (p<0.001). This finding highlights a pattern of increasing differences in community composition with distance (S7 Fig), albeit the relationship is not linear; rather, some degree of saturation can be seen in the graph, as at distances over 120 Km the dissimilarity appears to be stabilized.

**Fig 6:**
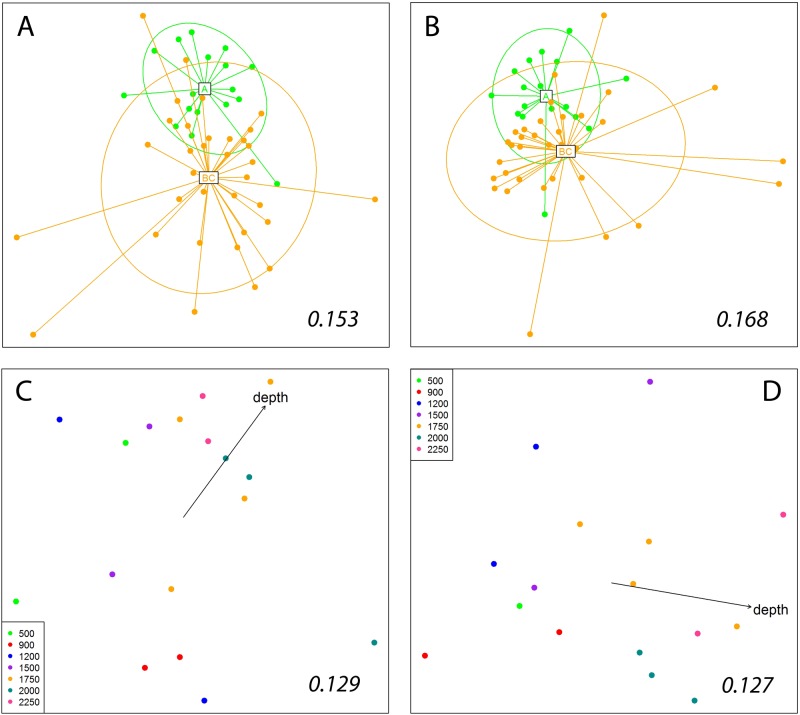
Non-metric Multidimensional Scaling plots of the sediment samples separated by layers (layer A, first cm of sediment in the corer; layer B, second cm; layer C, cm 3–5). Ordinations are obtained using Jaccard dissimilarity index (A), and Bray-Curtis dissimilarity index (B). The centroids of layer A and layers B+C, and the corresponding inertia ellipses, are indicated. (C) nmMDS plot obtained for layer A alone with the Jaccard index. (D) nmMDS plot obtained for layers B+C with the Jaccard index. Samples in these plots are presented with color-coded depth categories and the fitted gradient vector of depth is added. Numbers inside the plots are the stress values of the retained configurations.

Concerning the different layers of sediment (DOSMARES samples), PERMANOVA clearly showed differences between the most superficial layer A (first cm) and the two remaining layers (B, second cm) and C (cm 3 to 5), which were not different (Table 2). The factor Depth was also highly significant, but the interaction was not (Table 2), indicating similar effects of depth on the three layers. As before, qualitative and quantitative data yielded the same statistical outcomes, and only the former are presented in Table 2. The similarity between layers within the same mini-corer was 22.62±0.71 and 35.76±1.87% for the Jaccard and the Bray-Curtis coefficients, respectively (mean±SE). We subsequently plotted two groups (layer A and layers B+C) onto nmMDS ordinations (Fig 6). While there was some separation of the centroids, the differences seem to stem from a much higher heterogeneity (and hence dispersion in the nmMDS space) of samples in the layers B+C, a fact corroborated by a significant PERMDISP test (Table 2). nmMDS results from presence-absence and from abundance (n. of reads) data showed a highly similar arrangement (Fig 6, Procrustes correlation = 0.976, p<0.001).

**Table 2:** PERMANOVA analysis (9,999 permutations) comparing the factors Layer (fixed) and Depth (fixed) for the samples of the DOSMARES project using Jaccard index. PERMDISP probabilities for homogeneity of dispersion are also shown. Pairwise tests for levels of the significant factors are presented with uncorrected P-values. Significant values after FDR correction are indicated with an asterisk. The symbol § denotes comparisons for which PERMDISP detected a significant heterogeneity of dispersion (in comparisons of layers only, as the overall test was not significant for depth).

	*df*	*SS*	*pseudo-F*	*P-value*	*Permdisp*
Layer	2	9,053.3	1.565	0.004	0.026
Depth	6	30,412	1.752	<0.001	0.174
Layer*Depth	12	32,563	0.938	0.888	
Residual	30	86,775			
**Layer**					
Comparison	*t*	*P-value*			
A-B	1.360	0.006*^§^			
A-C	1.431	0.002*^§^			
B-C	0.935	0.704			
**Depth**					
Comparison	*t*	*P-value*			
500–900	1.214	0.158			
500–1200	1.168	0.201			
500–1500	1.214	0.155			
500–1750	1.477	0.001*			
500–2000	1.638	0.003*			
500–2250	1.455	0.020			
900–1200	1.143	0.238			
900–1500	1.192	0.175			
900–1750	1.444	0.001*			
900–2000	1.563	0.002*			
900–2250	1.543	0.013*			
1200–1500	1.033	0.465			
1200–1750	1.250	0.024			
1200–2000	1.493	0.005*			
1200–2250	1.381	0.032			
1500–1750	1.060	0.311			
1500–2000	1.336	0.018			
1500–2250	1.290	0.074			
1750–2000	1.209	0.037			
1750–2250	1.255	0.018			
2000–2250	1.169	0.124			

The significant depth effect was not due to differences in heterogeneity of dispersion, and pairwise comparisons showed significant or marginally significant effects when layers separated by several depth levels were compared (Table 2). When the samples for layer A and for layers B+C were plotted separately, the patterns obtained were significantly correlated (Procrustes correlation = 0.702, p<0.001), and a gradient of ordination by depth emerged in both cases (Fig 6). When depth was fitted as an environmental vector, it was significantly correlated with the ordinations obtained (*r* ^2^ = 0.612, p = 0.002 and *r* ^2^ = 0.675, p<0.001 for layers A and B+C, respectively). Again, ordinations obtained from presence-absence data (Jaccard index) and abundance data (Bray-Curtis index, not shown) yielded similar results (Procrustes correlations, p<0.001 in both cases), and the same occurred with the statistical analyses; thus only ordinations using the Jaccard index are shown in Fig 6.

### Analyses of main groups

The benthic MOTUs and the most MOTU-rich groups of metazoans (phyla Annelida, Arthropoda, and Nematoda) were analysed separately. We identified 742 MOTUs as benthic organisms. Their pattern of similarity among samples followed the same decreasing trend seen in the global dataset (S8 Fig). The same pattern was found for the major metazoan phyla, although in the case of annelids there was no decrease in the similarity when comparing between zones and between areas. In addition, in this group the similarity of mini-corers within the same box-corer was higher (48.02%) than in the global dataset (31.67%) or in the other phyla considered (less than 30%).

**Fig 7:**
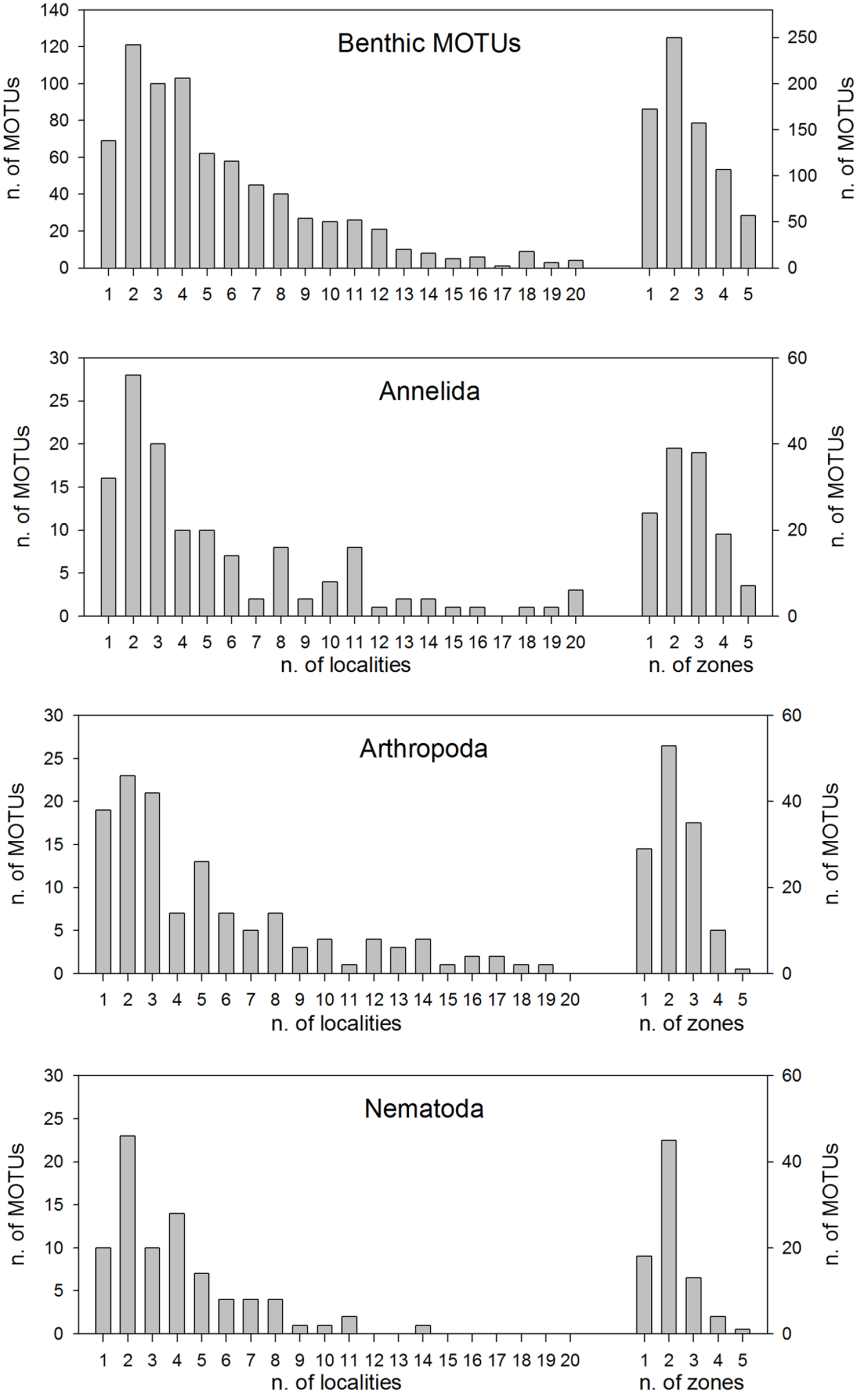
Distributional span of the MOTUs of the benthic taxa and the main metazoan phyla analysed. The graphs represent the frequency histograms of the number of MOTUs present in 1 to 20 samples (left axis), or in 1 to 5 zones (right axis).

The spatial distribution of the MOTUs in these groups (Fig 7) showed that in all cases the highest frequency of occurrence was at two localities (as in the global dataset) and the mean span was ca. 5.5 localities for benthic MOTUs and 5.3 for annelids and arthropods. Nematodes were the group less widespread, with each MOTU present in an average of 3.9 localities and only one MOTU present in more than 11 localities. Per zones, benthic MOTUs and nematodes had the highest frequency of occurrence at one or two zones, while annelids and arthropods tended to be more widespread (with a higher frequency of MOTUs common to two and three zones, Fig 7).

**Fig 8:**
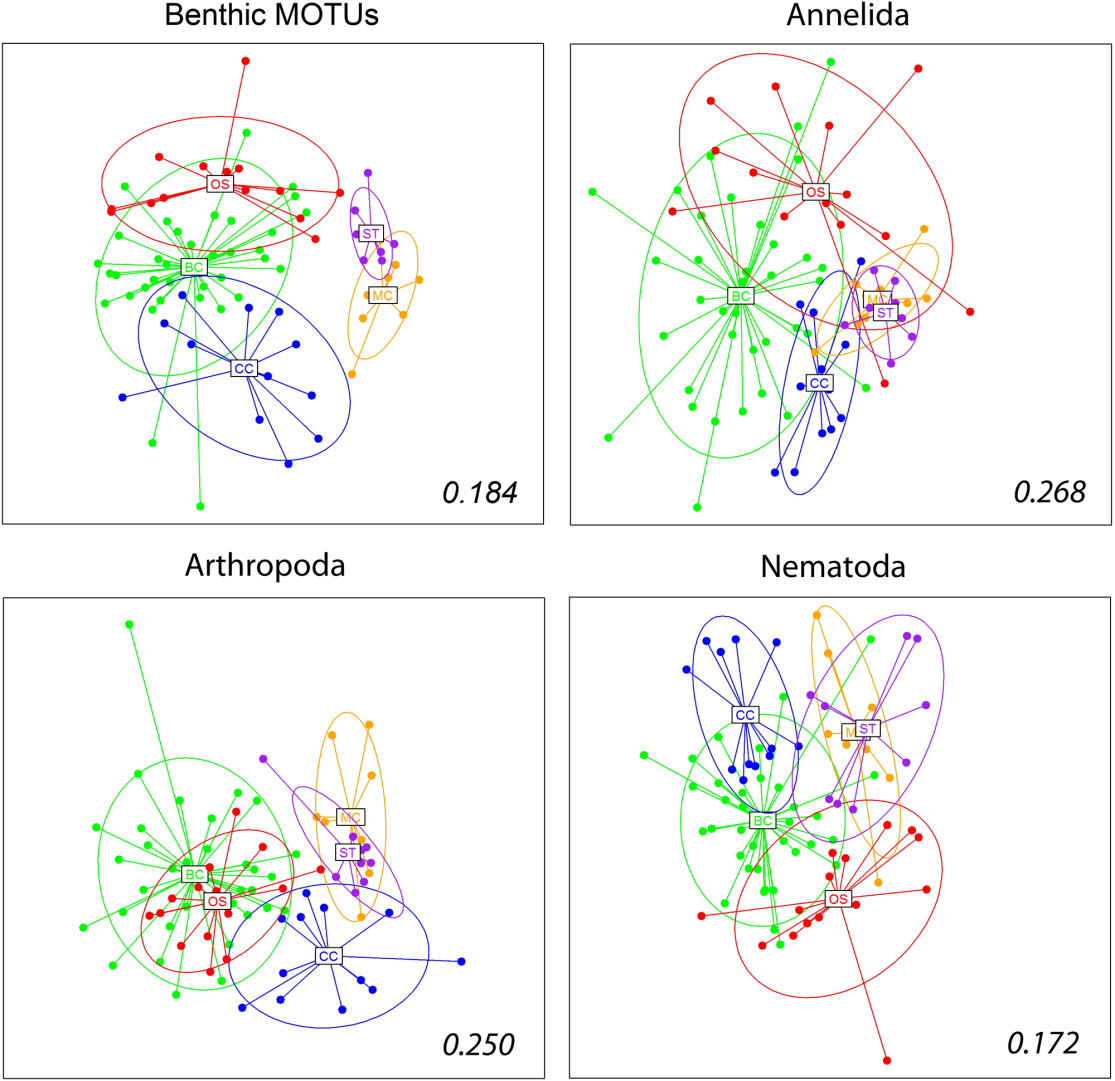
Non-metric Multidimensional Scaling plots of the samples obtained using Jaccard dissimilarity index for the benthic taxa and the main metazoan phyla. The centroids for the different zones, and the corresponding inertia ellipses, are indicated. Numbers inside the plots are the stress values of the retained configuration.

The nmMDS ordinations of the main groups (Fig 8) recovered a similar configuration as in the global dataset for benthic MOTUs, while the differentiation between zones appeared less distinct with the other groups, particularly the separation between the two zones in the Balearic Islands (Fig 8). Nevertheless, PERMANOVA analyses showed a significant effect of the factor zone in all cases (S2 Table), and an heterogeneous level of dispersion in benthic taxa, annelids and arthropods. The nested factor locality was also significant in all cases (S2 Table).

## Discussion

Using a procedure targeting short 18S sequences of extracellular DNA coupled with Illumina sequencing, we have assessed the composition of deep-sea sediment communities across a range of depths and geophysical conditions. A high α-diversity was detected, with over 1,600 MOTUs identified in samples from 20 localities, with several levels of replication. This allowed us to detect a fine-grained heterogeneity at small scales (within corer and within locality) and important differences in taxa composition between geographic areas. The rarefaction curves showed that saturation is not found in most samples, which is likely due to the complexity of the communities studied and the fact that small fragments of extracellular DNA can persist in the sediments. On the other hand, MOTU accumulation curves revealed that >30 samples are necessary to encompass the MOTU richness of the whole region studied. Thus, although our sampling effort and sequencing depth allowed structure assessment and community comparison, more replicates and/or bigger samples are necessary if a full characterization of the diversity is the goal. It is likely that the actual living community in the sediments is less complex, and analysing RNA or a longer fragment of DNA (that could degrade more quickly) can offer a complementary picture. Another potential shortcoming is the use of two preservation methods (DESS and ethanol) in one of the cruises, which was related to logistics of the different projects. DESS is nowadays a proven method for preserving adequately the DNA in sediment samples [8, 9, 72]. In our case, no significant differences in number of reads or MOTUs was detected between replicates preserved in ethanol or in DESS, and no preservation-related structure could be seen in the analyses. We do not therefore expect to have any bias in the results due to the preservation method.

The use of a separation step (via elutriation) of organisms prior to DNA extraction affects the diversity of metazoan vs non-metazoan taxa recovered in metabarcoding of meiofaunal assemblages [45]. We chose to work on bulk samples unsieved and unsorted [39, 46, 47, 73] to retain all extracellular DNA. This procedure allowed us to obtain DNA originated in organisms that had been present in the sediments and in the water column. We identified a 28% of photosynthetic MOTUs in our deep sea samples, that should correspond to DNA imported from the water column, and only ca. half (46%) of our MOTUs could be assigned to exclusively benthic (or with short-lived pelagic larvae) organisms. The true percent of benthic organisms should be, notwithstanding, higher than that, as many MOTUs had long-lived larvae or could not be assigned an unambiguous lifestyle. Pawlowski et al. [6] reported the finding of >30% planktonic OTUs in sediment samples. Given the abundance and ubiquity of DNA in marine sediments [74, 75], our approach can provide a cost-effective way to characterize a large fraction of the marine biodiversity using the sediments as DNA repositories that keep record of organisms above and below the sediment interface. Further studies should be aimed at comparing the information gleaned from extraction methods with and without lysis step (thus capturing mostly intracellular or extracellular DNA, respectively), and from DNA and RNA, which potentially indicates the living organisms present at the time of collection (e.g. [12,73]).

One of the recognized problems of the metabarcoding approach is the number of artefacts and errors generated during the amplification and sequencing process, chimeric sequences among them [76–79]. We tried to avoid these shortcomings using a stringent filtering procedure. For instance, we eliminated not only singletons [79], but all sequences with less than 10 reads. We also discarded all reads with less than 90% similarity with their best hit in GenBank. The associated risk it that they may correspond to deeply divergent lineages [8, 39, 46] not adequately represented in the current databases. The ecotag procedure assigns sequences to a taxon that is the most recent common ancestor of the sequence in the database with the highest similarity (best hit) to the query sequence, and all other sequences in the database within this level of similarity to the best hit. This procedure therefore places robustly a sequence in a given taxonomic rank. When considering sequences whose best hit was <90%, however, ecotag could not assign a rank (other than Eukarya) to most (92%) of them; another 4% could be reliably placed within Metazoa, but of these the majority could not be assigned to a phylum. Lacking a robust taxonomic placement, we preferred not to use these sequences. The compromise between reliability and efficiency is not easy to reach [62], and the need for comprehensive reference databases should be stressed, in particular for deep-sea communities. Any metabarcoding dataset is as good as the reference database is. In general, we have taken conservative options at the several steps involved in the analysis. We consider, then, than the final dataset is reliable.

Another aspect that needs to be taken into consideration is the potential of metabarcoding for quantitative analyses [5, 66]. Assessing abundance of organisms using read number is risky and needs previous calibration, as the number of reads will depend on the size of an organism, on differences in rDNA copy number among groups [3, 80], and on the potential primer bias (leading to unequal amplification success in different taxa). Only in particular cases can inferences about species abundance be made from read abundances [65]. Relative abundance values may be more reliable [6, 13]. For many comparative purposes it suffices that differences in relative abundances of a given group do reflect abundance shifts related, for instance, to habitat or season [80]. Some studies recommend to use only presence-absence metrics [66], while other works show that there is generally a correlation between read abundances and biomass of the different groups, so quantitative information, even if only approximate, can be obtained from metabarcoding data [14,81,82]. Overall, obtaining quantitative estimates from eDNA remains challenging due to the many factors affecting the direct relationship between number of reads and biomass [5,35,82]. In our case, our rankings considering number of MOTUs and considering relative number of reads showed discrepancies that were related to the size of organisms (and hence biomass), so both variables reflect different aspects of the community composition. On the other hand, the β-diversity results and the community analyses yielded in general similar results when considering presence-absence and abundance data. Overall, the usefulness of quantitative data as a proxy for biomass in metabarcoding studies deserves further targeted investigation.

Metazoa was the most MOTU-rich and read-abundant group in our samples, followed by Alveolata (chiefly dinoflagellates and ciliates). Polychaeta, Crustacea, and Nematoda were dominant in number of MOTUs among the metazoans. While polychaetes are often the dominant group in macrofauna (e.g. [7, 83]), nematodes are the most abundant group in meiofaunal assemblages in general [38, 41, 84]. In our samples, however, Nematoda ranked third in total MOTU richness, and seventh among Metazoa in relative read abundance. The latter is expectable given than the number of reads may be down weighted by their small size. In other metabarcoding studies nematodes dominated the meiofauna [8, 9], although not necessarily at all sites or times [10, 16]. It should be noted that in all these works the meiofaunal fraction was physically separated prior to extraction of DNA directly from organisms, while we used unsieved samples and extracellular DNA. We had, therefore, DNA from organisms of all size fractions, not just meiofauna. Our primers were adequate for nematodes, as checked with the available databases, so theoretically there should be no amplification bias against this group. Our method of extraction, however, may be less effective at obtaining DNA from nematodes. A comparison with studies addressed specifically at the meiofaunal size fraction or with different extraction methods (with and without lysis step) will allow an in-depth comparison of the advantages and drawbacks of the different procedures.

We found a remarkable heterogeneity in community composition among localities, zones, and areas. The species turnover (β-diversity) is accordingly high; the number of shared MOTUs was below 30% when we compared localities within a given zone, and below 15% among areas. High microgeographical structure has been also found in other studies using metabarcoding of marine sediments [8]. This correlates also with structure shown in nmMDS configurations and PERMANOVA analyses, where only the two zones of the Balearic Islands did not appear as significantly different. Surprisingly, when we analysed MOTUs with supposedly restricted dispersal (benthic), the pattern of differentiation was very similar to the one obtained with the whole dataset (including DNA from planktonic and widely dispersing organisms). This is likely because our study area is split by two permanent hydrographic density fronts: the Catalan front over the Iberian Peninsula slope, and the Balearic front over the Balearic Islands slope [85]. This double barrier is probably unpassable by small pelagic organisms, and as a result both benthic and pelagic organisms in our samples tended to be distributed over a relatively restricted number of study zones.

When we analysed separately the three main metazoan groups, the overall picture obtained is similar to the one found with the general dataset. However, the annelids and arthropods (which comprise a mixture of planktonic, meroplanktonic, and benthic stages) appear less spread and, particularly, nematodes (lacking free-swimming stages) span in general one or two zones. Although the factor zone was significant in explaining the distribution of all these groups, the spatial ordinations showed less distinct clusters when the metazoan groups were considered separately.

We found differences in community structure between sediment layers, in particular between the more superficial and the two subjacent layers, which were not significantly different. The deeper layers seemed to act as a sink of DNA, harbouring a more heterogeneous community. It is possible that marine sediments behave in a way similar to terrestrial soils, where DNA progressively leaches towards deeper layers [86]. A recent metabarcoding work in the Northern Gulf of Mexico [16] documented changes in composition of the first 3 cm of sediment corers with respect to the deeper (3–10 cm) layer, and this difference varied temporally and spatially.

The drivers of community structure in deep-sea bottoms are diverse, and topographic features and organic matter input (linked to primary productivity of overlying waters) are usually highlighted [87–89]. In the area studied a further driver is the bottom trawling fishery [38, 48], which reaches down to 1000 m [90] and has effects on sediments much below that depth [50]. The marked heterogeneity at several levels detected with our molecular methods is consistent with spatial heterogeneity found in morphology-based studies of several components (from megafauna to meiofauna) of deep-sea bottoms in the study area or in adjacent zones [41, 91–93]. Our results indicate a differentiation in community structure among depths when considering both presence-absence and relative abundance (in number of reads) data. However, we did not find any significant difference in MOTU richness with depth, which can be partly a result of mixing the DNA from benthic and planktonic origins. If any, richness was lower at the shallowest localities (500 m). In general, diversity is higher at intermediate depths (i.e., the slope area) in deep-sea communities (e.g. [94, 95], although the pattern is not universal [38]. In the Blanes Canyon, a peak in biomass has been observed at intermediate (1050–1350 m) depths for megafaunal biomass [92], but no clear trends with depth were observed (or they were detected only seasonally) when considering meiofaunal components [84, 96].

Deep-sea environments cover more than 65% of the earth’s surface and support key ecosystem services, yet they are amongst the least known habitats on earth. It has been estimated that less than 1% of deep-sea species are presently described [8, 37, 97]. Although there remain a number of biases and pitfalls to be addressed (e.g. [3, 79, 98, 99]), metabarcoding can produce comprehensive biodiversity datasets in a short time frame. Morphological approaches are invaluable, but they are more time-consuming and require taxonomic expertise often difficult to obtain. Recent works have shown that molecular results compare well with high quality datasets obtained by conventional methods [11, 24, 100, 101]. We have shown how a metabarcoding approach can provide community characterization and α- and β-diversity values for deep-sea environments, which are the raw materials on which ecology and management build. The field of metabarcoding holds promise for efficiently moving from ecosystem assessment to ecosystem management [102]. Instead of using a subset of taxa as indicator or umbrella species, we advocate the use of DNA repositories in bulk sediment samples for an efficient assessment of deep-sea biodiversity, which, combined with an appropriate presentation and communication strategy to end-users, will allow a fast transfer of information from monitoring to management of these invaluable environments.

## Conclusions

We applied a protocol optimized for the extraction of extracellular DNA to samples of deep-sea sediments. We assayed a new primer set that amplifies a short hypervariable region of the 18S rRNA gene in eukaryotes.

Our results uncovered high α- and β-diversity in the communities analysed. Using stringent filtering steps, we have identified 1,629 Molecular Operational Taxonomic Units, with Metazoa being the most diverse group, followed by Alveolata, Stramenopiles, and Rhizaria. Among Metazoa, Arthropoda, Annelida, and Nematoda were the most diverse phyla present in the samples.

Heterogeneity was present at all scales analysed: between sediment layers, between different corers, within and between localities, and between geographical zones studied.

Overall, results obtained from qualitative (presence/absence) and quantitative (relative abundance of reads) data showed similar patterns of community structure.

The bottoms studied are of great ecological and economic value, and face important threats nowadays. Our results show that metabarcoding can be used to accurately assess the biodiversity and community differentiation of these bottoms and set the ground for future monitoring and conservation efforts.

## Supporting Information

S1 Fig Map of the Western Mediterranean, with detail of the sampled area. CC, Cap de Creus Canyon; BC, Blanes Canyon; OS, Blanes Open Slope; MC, Menorca Canal; ST, Serra de Tramuntana Slope.
(TIF)

S2 Fig Characteristics of the 18S_allshorts primer pair targeting eukaryotic groups. A) Primer logos according to all eukaryote sequences of the EMBL database (release 117); B) Number of mismatches of the forward and reverse primers according to all eukaryote and non-eukaryote sequences of the EMBL database (release 117); note that virtually no non-eukaryotes appear in the figure, indicating that they have above 3 mismatches, which means that the primer pair is highly specific of eukaryotes; C) Length distribution of the amplicons (excluding primers).
(TIF)

S3 Fig Cumulative frequency of MOTUs with a given % identity with the best hit in the reference database. Only MOTUs with >90% similarity with best match. Values are shown separately for all MOTUs and for the main groups.
(TIF)

S4 Fig Proportions in the different localities of the number of MOTUs of the Super-groups and the main metazoan phyla considered. Codes of localities as in Table 1.
(TIF)

S5 Fig Rarefaction curves. The number of MOTUs obtained at increasing number of reads for each sample are indicated.
(TIF)

S6 Fig MOTU accumulation curves obtained pooling samples together. Grey areas represent 95% confidence intervals obtained through randomization.
(TIF)

S7 Fig Plot of the geographic distance versus community dissimilarity measures obtained with the Jaccard index. (TIF)

S8 Fig Average similarities between pairs of samples in different categories of comparisons. The benthic MOTUs and the main metazoan groups are presented separately. Values correspond to the Jaccard index (presence-absence data). Error bars are standard errors.
(TIF)

S1 File Choice of threshold value for clustering. (DOC)

S1 Table Details of the localities sampled in the two cruises. (DOCX)

S2 Table PERMANOVA analysis (9,999 permutations) comparing the five zones surveyed for qualitative data of the benthic MOTUs and the main metazoan groups. Note that one (Nematoda) and two (Arthropoda) samples were removed from analyses because they had too few reads and appeared as clear outliers. PERMDISP probabilities for homogeneity of dispersion are also shown.
(DOCX)
