# Correction: Deep-Sea, Deep-Sequencing: Metabarcoding Extracellular DNA from Sediments of Marine Canyons

**DOI:** 10.1371/journal.pone.0153836

**Published:** 2016-04-13

**Authors:** Magdalena Guardiola, María Jesús Uriz, Pierre Taberlet, Eric Coissac, Owen Simon Wangensteen, Xavier Turon

There is an error in the third sentence of the second paragraph under the “DNA extraction, amplification and next generation sequencing” subheading of the Materials and Methods section. The correct sentence is: The primer pair was labelled 18S_allshorts (Forward 5’-TTTGTCTGSTTAATTSCG-3’ and Reverse 5’-TCACAGACCTGTTATTGC -3’).
