# Supplementary figures and images for: Deep-Sea, Deep-Sequencing: Metabarcoding Extracellular DNA from Sediments of Marine Canyons
Source: PLoS One. 2015 Oct 5;10(10):e0139633. doi: 10.1371/journal.pone.0139633 (PMC4593591; doi:10.1371/journal.pone.0139633)

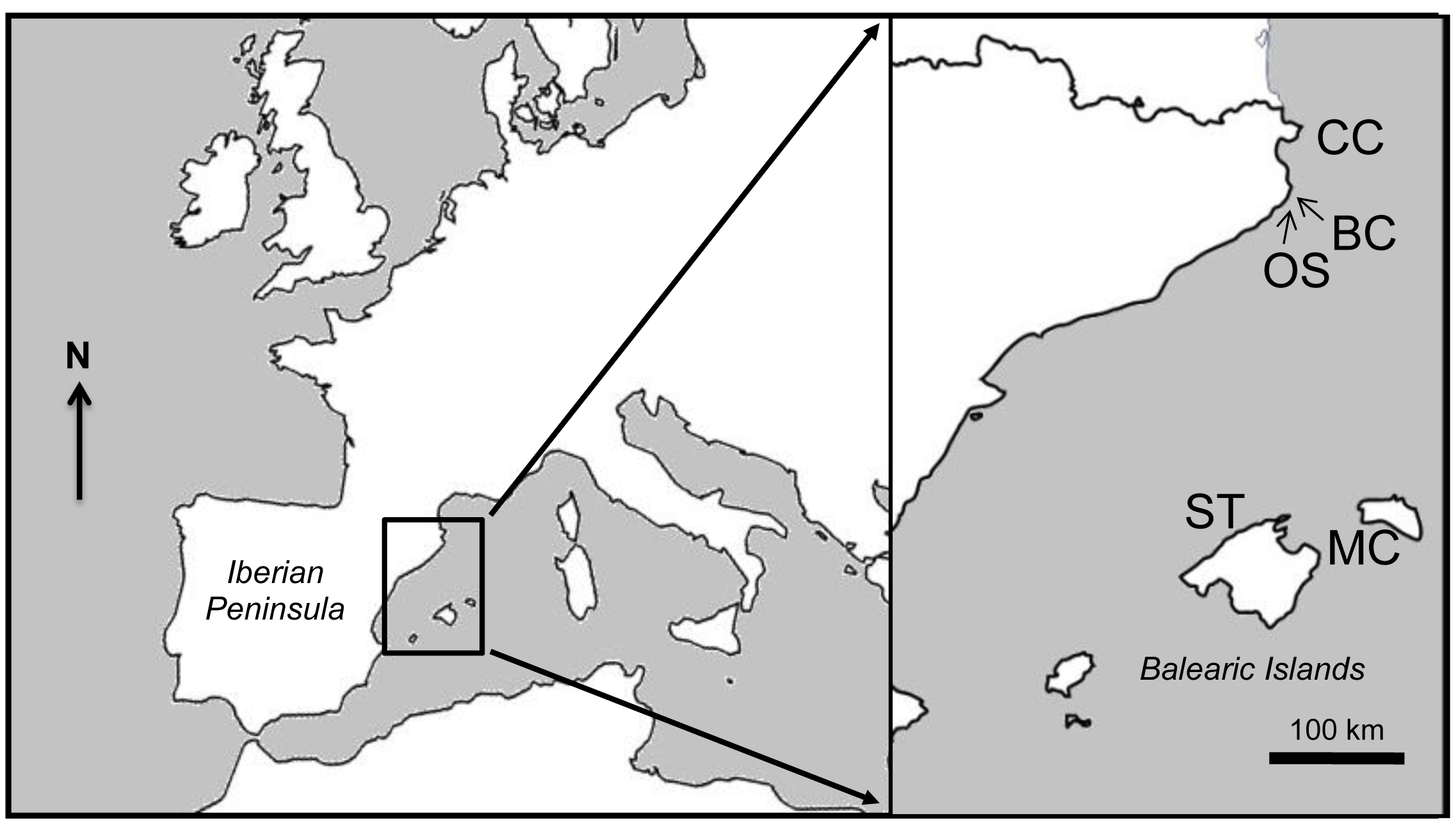

Supplement: S1 Fig — CC, Cap de Creus Canyon; BC, Blanes Canyon; OS, Blanes Open Slope; MC, Menorca Canal; ST, Serra de Tramuntana Slope. (TIF) [file pone.0139633.s001.tif]

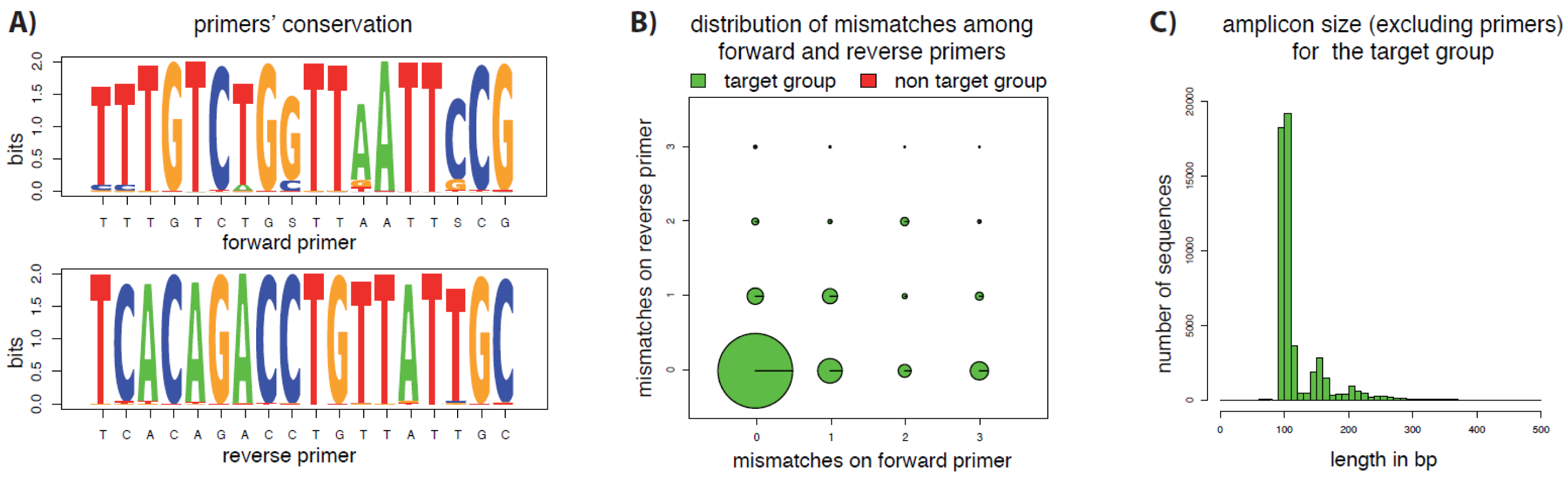

Supplement: S2 Fig — A) Primer logos according to all eukaryote sequences of the EMBL database (release 117); B) Number of mismatches of the forward and reverse primers according to all eukaryote and non-eukaryote sequences of the EMBL database (release 117); note that virtually no non-eukaryotes appear in the figure, indicating that they have above 3 mismatches, which means that the primer pair is highly specific of eukaryotes; C) Length distribution of the amplicons (excluding primers). (TIF) [file pone.0139633.s002.tif]

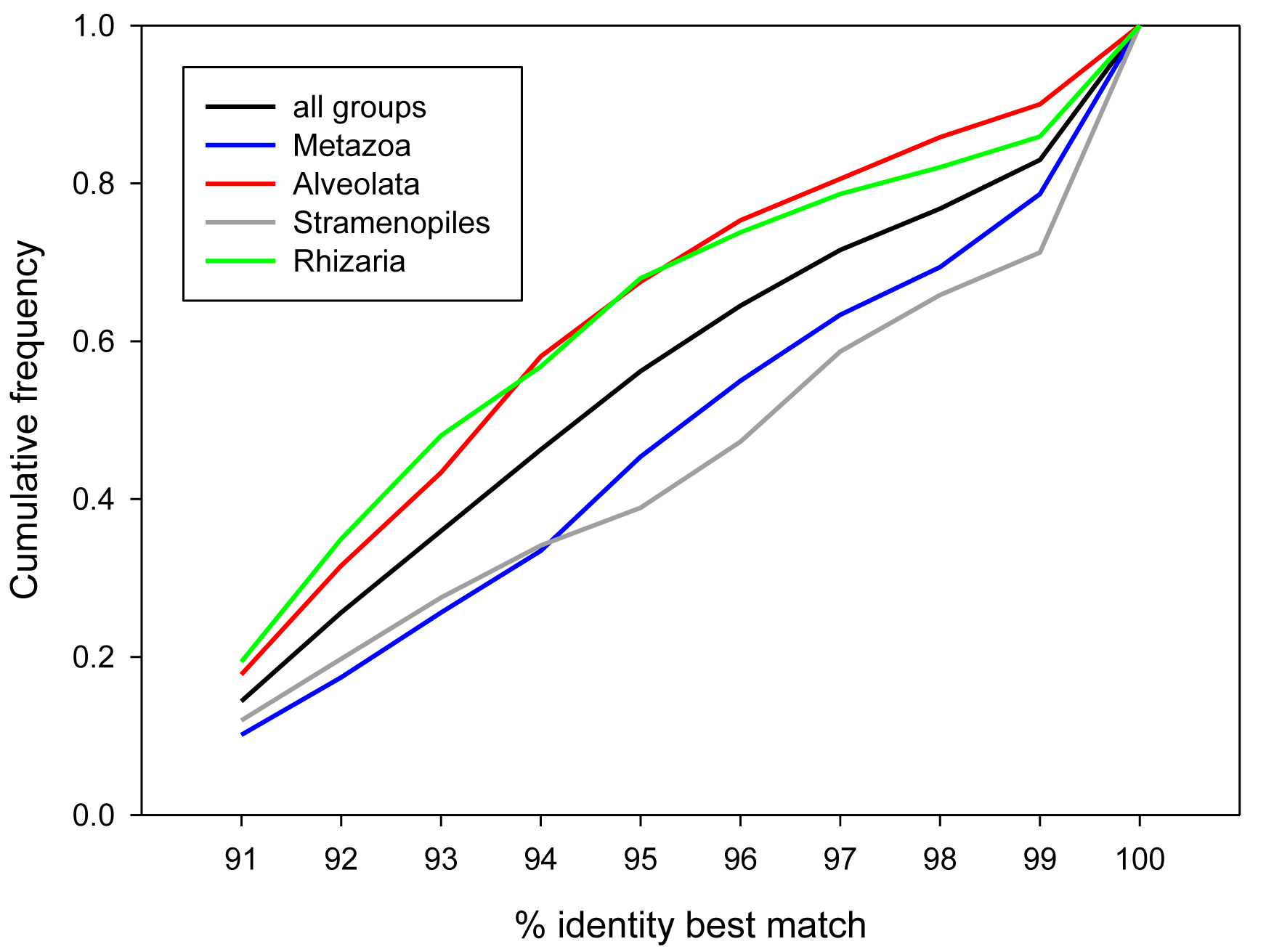

Supplement: S3 Fig — Only MOTUs with >90% similarity with best match. Values are shown separately for all MOTUs and for the main groups. (TIF) [file pone.0139633.s003.tif]

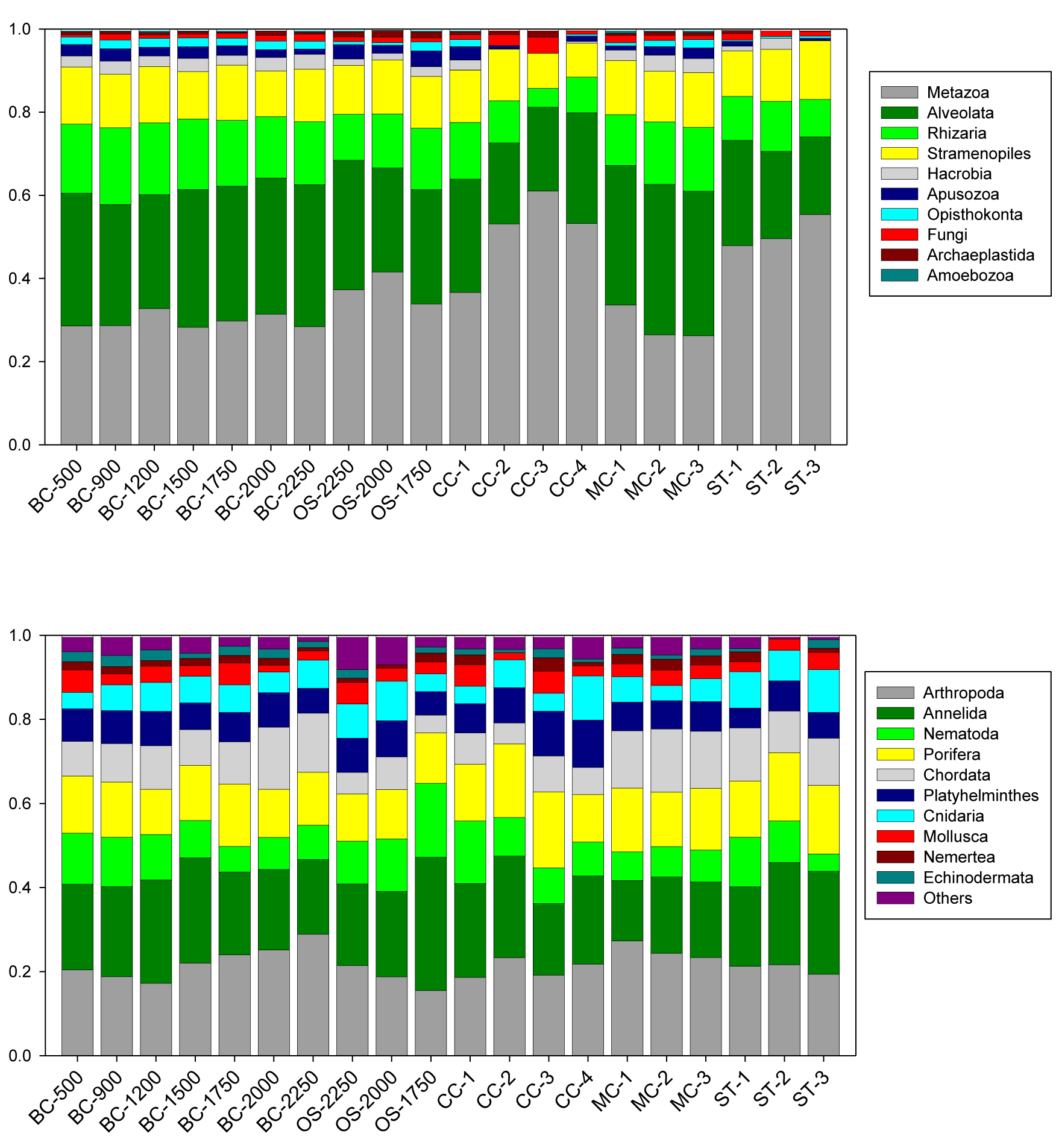

Supplement: S4 Fig — Codes of localities as in Table 1. (TIF) [file pone.0139633.s004.tif]

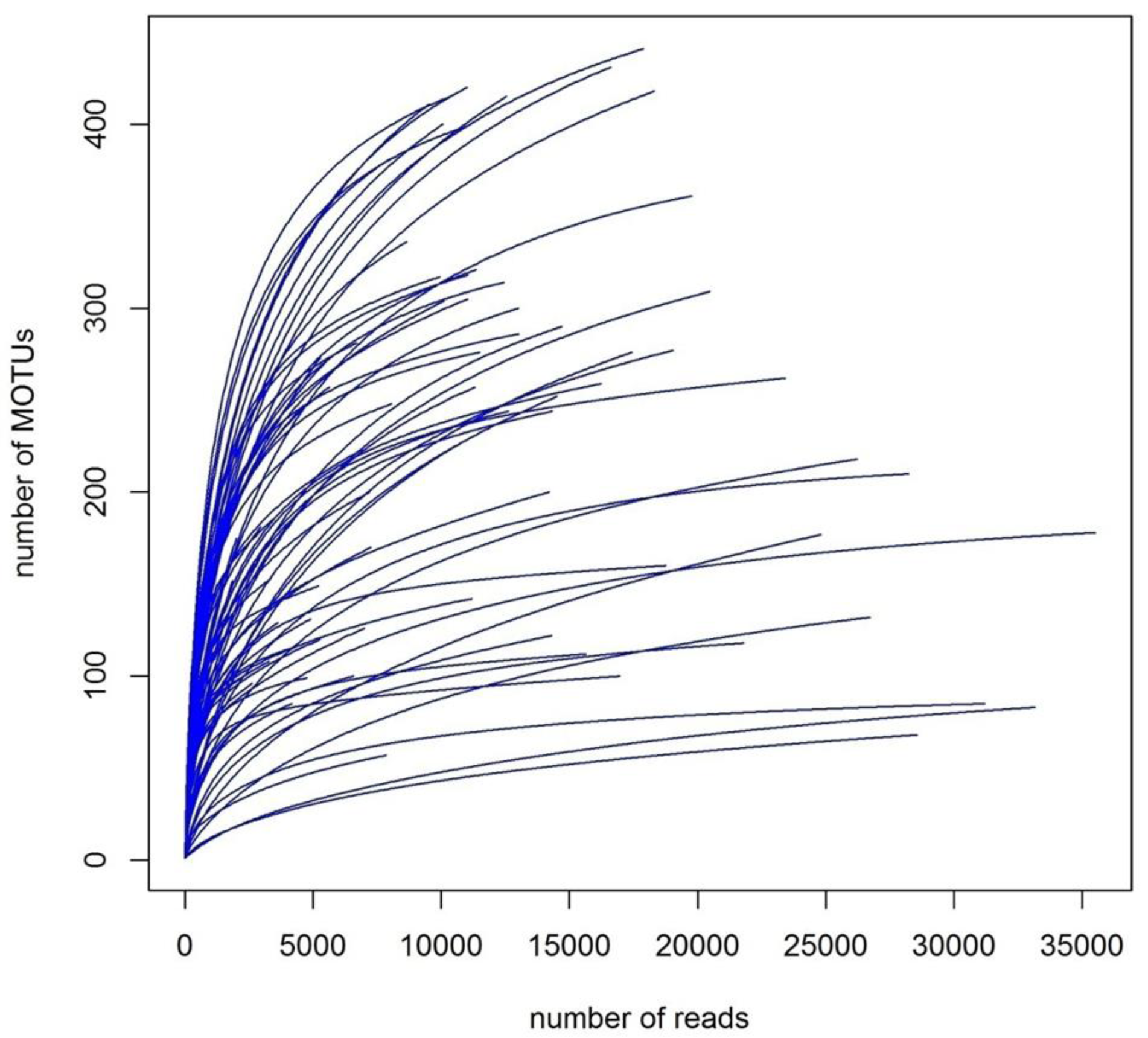

Supplement: S5 Fig — The number of MOTUs obtained at increasing number of reads for each sample are indicated. (TIF) [file pone.0139633.s005.tif]

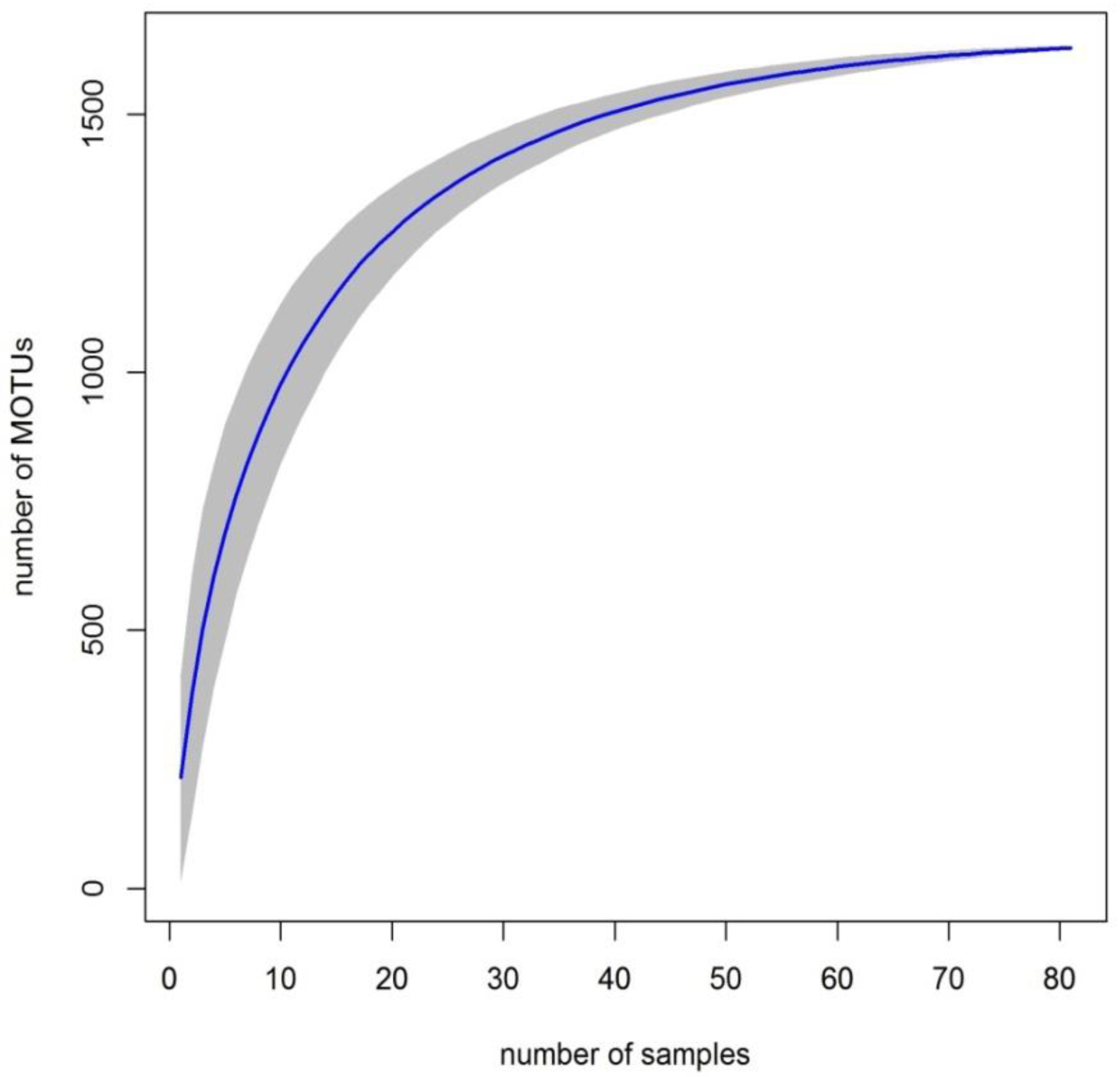

Supplement: S6 Fig — Grey areas represent 95% confidence intervals obtained through randomization. (TIF) [file pone.0139633.s006.tif]

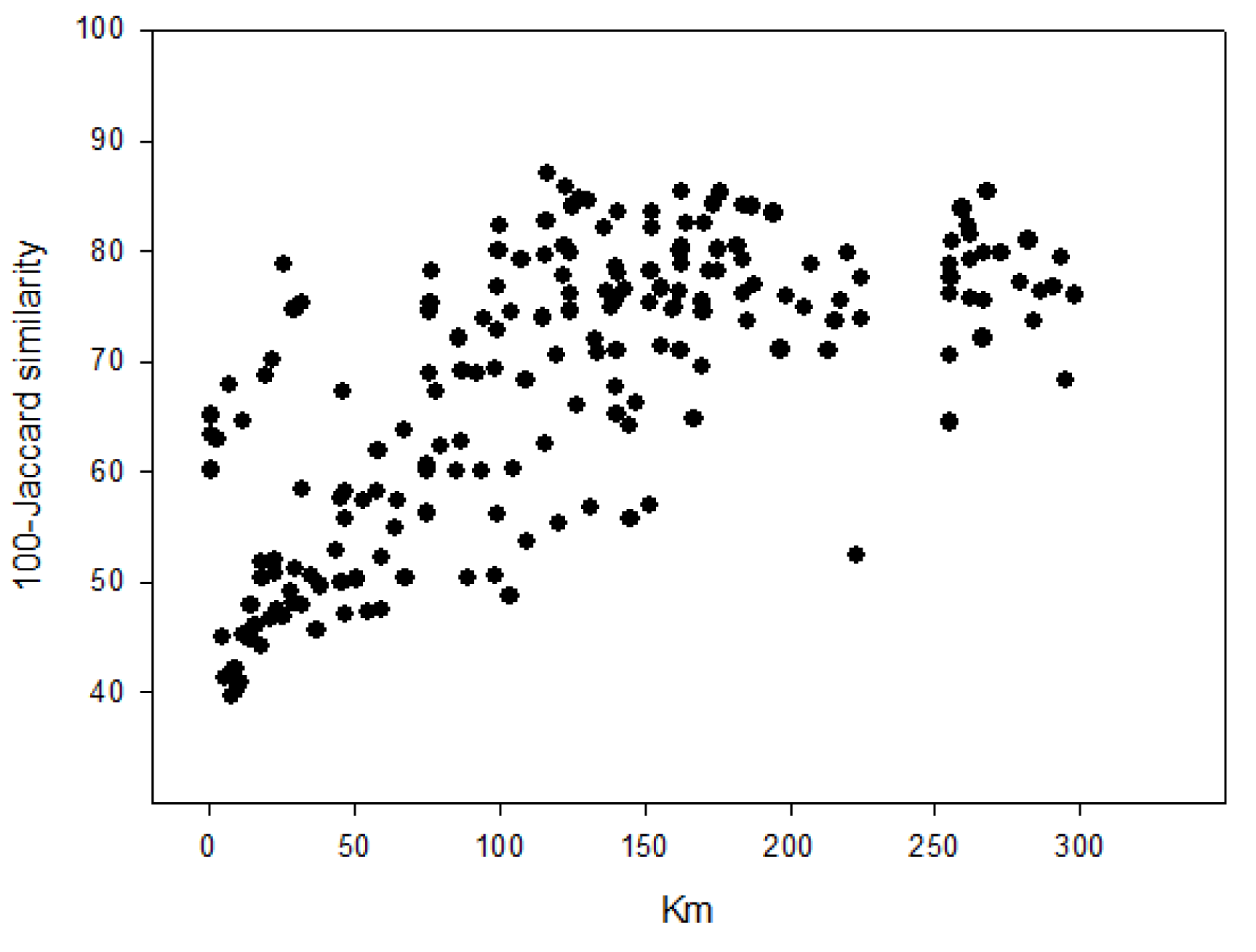

Supplement: S7 Fig — (TIF) [file pone.0139633.s007.tif]

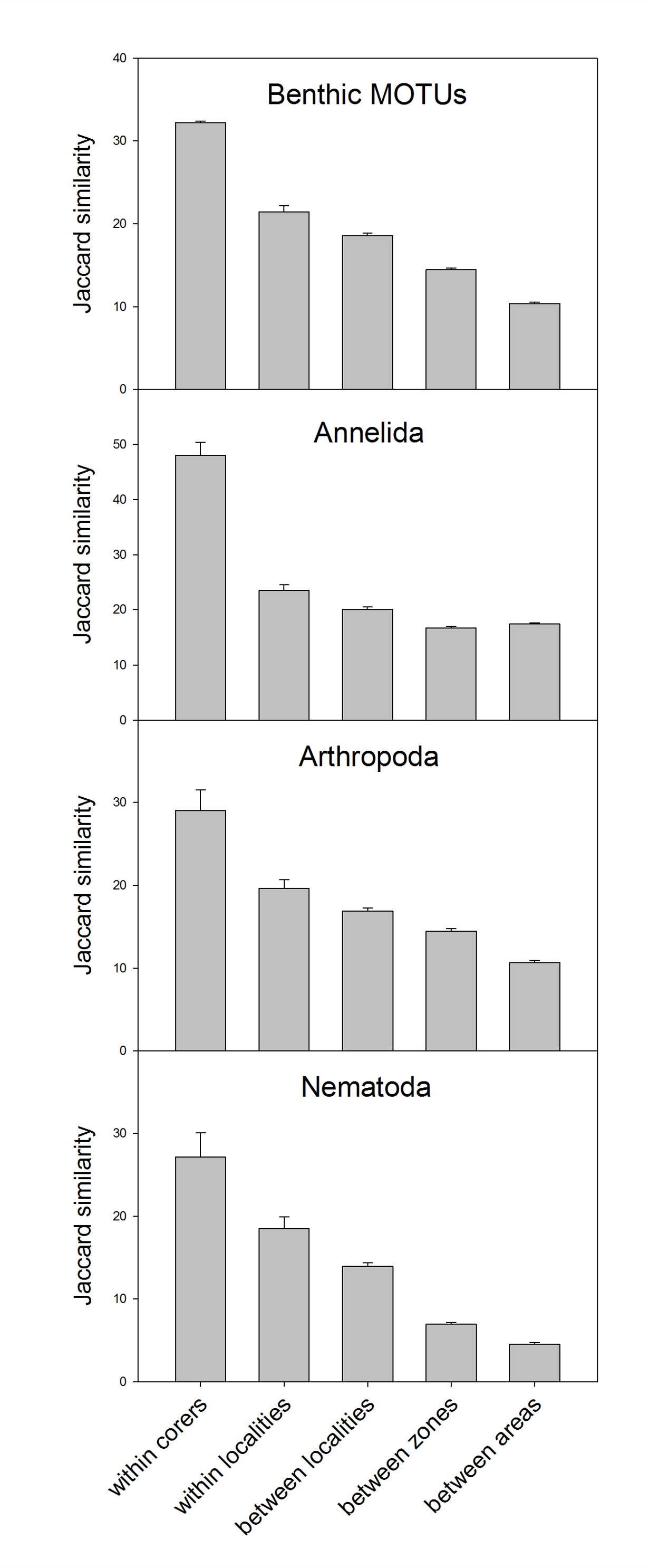

Supplement: S8 Fig — The benthic MOTUs and the main metazoan groups are presented separately. Values correspond to the Jaccard index (presence-absence data). Error bars are standard errors. (TIF) [file pone.0139633.s008.tif]
