# Supplementary material for: Deep-Sea, Deep-Sequencing: Metabarcoding Extracellular DNA from Sediments of Marine Canyons
Source: PLoS One. 2015 Oct 5;10(10):e0139633. doi: 10.1371/journal.pone.0139633 (PMC4593591; doi:10.1371/journal.pone.0139633)
Supplement: S1 File — (DOC) [file pone.0139633.s009.doc]

**S1 File**

Finding the adequate threshold for clustering is a contentious matter [1]. The ideal similarity value is the one that more closely approximates the species-level diversity of a sample. The choice is always a compromise between an inflation of taxonomic units below the species level and an underestimation of the true species diversity through lumping sequences into higher-level groups. Besides, a single cut-point is highly unlikely to suit all groups in a complex sample [2, 3].

The distribution of number of MOTUs in our dataset at increasing levels of similarity (Fig. 1) did not show any abrupt change of slope that could suggest a barcode gap (i.e. a gap between intra- and interspecific similarities). Rather, there was a steady increase in the number of clusters recovered.

**Fig. 1. Number of clusters generated as a function of % similarity using our dataset**

Porazinska et al.[4] assembled an artificial nematode community comprising 41 species sequenced for a variable region (v7-v8) of the 18S rRNA gene encompassing our target sequence. Using this dataset, Porazinska et al. [5] found that a 95% threshold would recover 68% of the species known to be present. A 99% threshold recovered all species, but inflated (18-fold) the number of MOTUs present, requiring *a posteriori* filtering using the frequency structure of the MOTUs belonging to the same species. Using that same control community, a value of 96% was afterwards suggested as a conservative value that effectively emulates species richness for estimating MOTUs in meiobenthic communities, and was applied to sequences of the v1-v2 region of the 18SrRNA gene [6,7]. Lallias et al. [8] also used this 96% threshold for the v1-v2 region of the 18S rRNA gene and found good correlation of nematode richness with previous morphological studies. Sometimes, a second cut-off point of 99% has been used to estimate genotypic, rather than species, diversity [7]. It should be noted that the v1-v2 region of the 18S rRNA gene is in general more variable that the v7-v8 region [9]. Bik et al. [10], using two regions of the gene (v1-v2 and v7-v8) adopted two similarity thresholds: 95% (relaxed clustering) and 99% (stringent clustering). Dafforn et al. [11] amplified the v7 region (slightly longer segment than the one used in our study) and applied a 97% cut-off value that slightly overestimated biodiversity, as judged by control assemblages.

Considering the above results, we chose a 96% clustering threshold for our samples. This is likely to be on the conservative side, so we are possibly underestimating to some degree the true species-level biodiversity present, and this degree is likely variable for different taxonomic groups. However, given our goal to assess the feasibility and efficiency of metabarcoding methods targeting extracellular DNA, we prefer to adopt a conservative approach rather than risking an inflation of MOTUs not reflecting adequately species distribution patterns.

**References**

1. Chen W, Zhang CK, Cheng Y, zhang S, Zhao H (2013) A comparison of methods for clustering 16S rRNA sequences into OTUs. PLoS One 8(8): e70837
2. Creer S, Fonseca G, Porazinska DL, Giblin-Davis RM, Sung W, et al. (2010) Ultrasequencing of the meiofaunal biosphere: practice, pitfalls and promises. Molecular Ecology 19:4-20
3. Brown EA, Chain FJJ, Crease TJ, MacIsaac HJ, Cristescu ME (2015) Divergence thresholds and divergent biodiversity estimates: can metabarcoding reliably describe zooplankton communities? Ecology and Evolution online version. DOI: 10.1002/ece3.1485
4. Porazinska DL, Giblin-Davis RM, Faller L, Farmerie W, Kanzaki N, et al. (2009) Evaluating high-throughput sequencing as a method for metagenomic analysis of nematode diversity. Molecular Ecology Resources 9:1439-1450
5. Porazinska DL, Giblin-Davis RM, Sung W, Thomas WK (2010) Linking operational clustered taxonomic units (OCTUs) from parallel ultra sequencing (PUS) to nematode species. Zootaxa 2427:55-63
6. Fonseca VG, Carvalho GR, Sung A, Johnson HF, Power DM, et al. (2010) Second-generation environmental sequencing unmasks marine metazoan biodiversity. Nature Communications 1:8 pp
7. Fonseca VG, Carvalho GR, Nichols B, Quince C, Johnson HF, et al. (2014) Metagenetic analysis of patterns of distribution and diversity of marine meiobenthic eukaryotes. Global Ecology and Biogeography 23:1293-1302
8. Lallias D, Hiddink JG, Fonseca VG, Gaspar JM, Sung W, et al. (2014) Environmental metabarcoding reveals heterogeneous drivers of microbial eukaryote diversity in contrasting estuarine ecosystems. 1-14
9. Hadziavdic K, Lekang K, Lanzen A, Jonassen I, Thompson EM, et al. (2014) Charecterization of the 18S rRNA gene for designing universal eukaryote specific primers. PLoS One 9(2): e87624
10. Bik HM, Sung W, De Ley P, Baldwin JG, Sharma J, et al. (2012) Metagenetic community analysis of microbial eukaryotes illuminates biogeographic patterns in deep-sea and shallow water sediments. Molecular Ecology 21:1048-1059
11. Dafforn KA, Baird DJ, Chariton AA, Sun MY, Brown MV, et al. (2014) Faster, higher, and stronger? The pros and cons of molecular faunal data for assessing ecosystem condition. Advances in Ecological Research 51
