# Supplementary material for: Deep-Sea, Deep-Sequencing: Metabarcoding Extracellular DNA from Sediments of Marine Canyons
Source: PLoS One. 2015 Oct 5;10(10):e0139633. doi: 10.1371/journal.pone.0139633 (PMC4593591; doi:10.1371/journal.pone.0139633)
Supplement: S1 Table — (DOCX) [file pone.0139633.s010.docx]

S1 Table.

| **Project DOSMARES** | | | | | | |
| --- | --- | --- | --- | --- | --- | --- |
| **Zone** | **Date** | **Locality** | **Depth(m)** | **Lat. N** | **Long. E** |  |
| Blanes Canyon (BC) | 11/03/2012 | BC-500 | 477 | 41º34'28" | 41º34'28" |  |
|  | 13/03/2012 | BC-500 | 472 | 41º34'28" | 41º34'28" |  |
|  | 13/03/2012 | BC-900 | 866 | 41º34'28" | 41º34'28" |  |
|  | 13/03/2012 | BC-900 | 835 | 41º34'28" | 41º34'28" |  |
|  | 13/03/2012 | BC-1200 | 1,210 | 41º34'28" | 41º34'28" |  |
|  | 13/03/2012 | BC-1200 | 1,250 | 41º34'28" | 41º34'28" |  |
|  | 14/03/2012 | BC-1500 | 1,487 | 41º34'28" | 41º34'28" |  |
|  | 14/03/2012 | BC-1500 | 1,485 | 41º34'28" | 41º34'28" |  |
|  | 14/03/2012 | BC-1750 | 1,748 | 41º34'28" | 41º34'28" |  |
|  | 14/03/2012 | BC-1750 | 1,748 | 41º34'28" | 41º34'28" |  |
|  | 14/03/2012 | BC-2000 | 1,972 | 41º34'28" | 41º34'28" |  |
|  | 14/03/2012 | BC-2200 | 2,211 | 41º34'28" | 41º34'28" |  |
| Blanes Open Slope (OS) | 14/03/2012 | OS-2250 | 2,222 | 40º54'20" | 03º12'19" |  |
|  | 15/03/2012 | OS-2000 | 1,975 | 41º03'05" | 03º01'22" |  |
|  | 15/03/2012 | OS-2000 | 1,975 | 41º07'19" | 02º57'23" |  |
|  | 15/03/2012 | OS-1750 | 1,731 | 40º54'20" | 03º12'19" |  |
|  | 15/03/2012 | OS-1750 | 1,751 | 41º03'05" | 03º01'22" |  |
| **Project INDEMARES** | | | | | | |
| **Zone** | **Date** | **Locality** | **Depth(m)** | **Lat. N** | **Long. E** |  |
| Serra de Tramuntana Slope (ST) | 29/06/2012 | ST-1 | 370 | 40º00'33" | 03º08'11" |  |
|  | 29/06/2012 | ST-2 | 474 | 40º00'24" | 03º07'99" |  |
|  | 29/06/2012 | ST-3 | 544 | 40º00'42" | 03º07'87" |  |
| Menorca Canal (MC) | 01/06/2012 | MC-1 | 547 | 39º48'34" | 03º59'07" |  |
|  | 01/06/2012 | MC-2 | 800 | 39º46'68" | 03ª59'07" |  |
|  | 04/06/2012 | MC-3 | 137 | 39º56'98" | 03º28'59" |  |
| Cap de Creus  Canyon (CC) | 15/06/2012 | CC-1 | 128 | 42º17'42" | 03º28'09" |  |
|  | 16/06/2012 | CC-2 | 85 | 42º18'15" | 03º15'00" |  |
|  | 16/06/2012 | CC-3 | 94 | 42º22'02" | 03º15'00" |  |
|  | 17/06/2012 | CC-4 | 99 | 42º24'33" | 03º15'13" |  |
