# Supplementary material for: Deep-Sea, Deep-Sequencing: Metabarcoding Extracellular DNA from Sediments of Marine Canyons
Source: PLoS One. 2015 Oct 5;10(10):e0139633. doi: 10.1371/journal.pone.0139633 (PMC4593591; doi:10.1371/journal.pone.0139633)
Supplement: S2 Table — Note that one (Nematoda) and two (Arthropoda) samples were removed from analyses because they had too few reads and appeared as clear outliers. PERMDISP probabilities for homogeneity of dispersion are also shown. (DOCX) [file pone.0139633.s011.docx]

S2 Table.

| **BENTHIC MOTUs** | *df* | *SS* | *pseudo-F* | *P-value* | *Permdisp* |
| --- | --- | --- | --- | --- | --- |
| Zone | 4 | 53,367 | 3.242 | <0.001 | <0.001 |
| Locality(Zone) | 15 | 64,507 | 1.498 | <0.001 | <0.001 |
| Residual | 61 | 175,030 |  |  |  |
| **ANNELIDA** | *df* | *SS* | *pseudo-F* | *P-value* | *Permdisp* |
| Zone | 4 | 47,706 | 2.726 | <0.001 | <0.001 |
| Locality(Zone) | 15 | 69,840 | 1.872 | <0.001 | <0.001 |
| Residual | 61 | 151,710 |  |  |  |
| **ARTHROPODA** | *df* | *SS* | *pseudo-F* | *P-value* | *Permdisp* |
| Zone | 4 | 49,992 | 3.092 | <0.001 | <0.001 |
| Locality(Zone) | 15 | 62,741 | 1.430 | <0.001 | 0.004 |
| Residual | 59 | 172,610 |  |  |  |
| **NEMATODA** | *df* | *SS* | *pseudo-F* | *P-value* | *Permdisp* |
| Zone | 4 | 62,719 | 3.191 | <0.001 | 0.078 |
| Locality(Zone) | 15 | 77,207 | 1.604 | <0.001 | <0.001 |
| Residual | 60 | 192,550 |  |  |  |
